# ITGA4 as a potential prognostic and immunotherapeutic biomarker in human cancer and its clinical significance in gastric cancer: an integrated analysis and validation

**DOI:** 10.3389/fonc.2025.1513622

**Published:** 2025-02-12

**Authors:** Jiaxing Zhang, Gang Wang, Jie Liu, Futian Tang, Song Wang, Yumin Li

**Affiliations:** ^1^ The Second Hospital and Clinical Medical School, Lanzhou University, Lanzhou, China; ^2^ Digestive System Tumor Prevention and Treatment and Translational Medicine Engineering Innovation Center of Lanzhou University, Lanzhou University, Lanzhou, China; ^3^ School of Basic Medical Sciences of Lanzhou University, Lanzhou University, Lanzhou, China; ^4^ Ecosystem Change and Population Health Research Group, School of Public Health and Social Work, The Institute of Health and Biomedical Innovation, Queensland University of Technology, Brisbane, QLD, Australia

**Keywords:** ITGA4, pan-cancer, TME, immunotherapy, biomarker, gastric cancer

## Abstract

**Background:**

Integrin Subunit Alpha 4 (ITGA4), a member of the integrin protein family, is involved in the progression of malignant tumors. However, its role across different cancer types is not well understood.

**Methods:**

Utilizing multi-omics data, we comprehensively evaluated ITGA4’s expression, clinical relevance, diagnostic and prognostic value, functions, mutations, and methylation status, along with its impact on immunity, mismatch repair (MMR), heterogeneity, stemness, immunotherapy responsiveness, and drug resistance in pan-cancer, with partial validation in gastric cancer (GC) using transcriptomic analysis, single-cell data, western blot (WB), wound-healing assay, flow cytometry and immunohistochemistry (IHC). We further investigated its correlation with clinicopathology and serological markers on tissues from 80 GC patients.

**Results:**

ITGA4 expression was generally low in normal tissues but varied significantly across tumor types, with higher levels in advanced stages and grades. It demonstrated diagnostic value in 20 cancer types and effectively predicted 1-, 3-, and 5-year survival rates as part of a prognostic model. ITGA4 played roles in cell adhesion, migration, immune regulation, and pathways like PI3K-Akt and TSC-mTOR. It showed alterations in 22 cancer types, with methylation at 9 sites inhibiting its expression. ITGA4 positively correlated with immune cell infiltration, immune regulatory genes, chemokines, and might reduce microsatellite instability (MSI) and tumor mutation burden (TMB) by promoting MMR gene expression. It could also predict immunotherapy efficacy and chemotherapy sensitivity. In GC, high ITGA4 expression was related to poor prognosis, promoted tumor proliferation and migration, and enhanced immune cell infiltration. ITGA4 expression was higher in GC cells and tissues than normal ones. Its downregulation inhibited GC cell migration and promoted apoptosis. Moreover, ITGA4 was correlated with N stage, pathological stage, neural and vascular invasion, serum levels of Ki-67, immune cells, CRP and CA125.

**Conclusion:**

ITGA4 is a potential biomarker and therapeutic target to enhance cancer treatment and improve patient outcomes.

## 1 Introduction

Cancer significantly impacts public health and contributes to the global disease burden (1). According to WHO’s 2020 data, cancer is among the top causes of death before the age of 70 in most countries, with both incidence and mortality rates on the rise (2, 3). The Global Cancer Observatory (GLOBOCAN) notes that cancer causes nearly 10 million deaths each year (2). As global populations age, the mortality from cancer is expected to increase further (4). Cancer’s complexity arises from genetic and epigenetic changes in cells, enabling them to adapt and gain traits that enhance survival and proliferation, making treatment development more challenging (5). Traditional cancer treatments such as surgery, radiotherapy, and hormonal therapy are being complemented by innovative approaches like immunotherapy, gene therapy, and targeted molecular therapy (5). Additionally, advances in high-throughput sequencing and accessible public databases are enabling detailed studies of gene involvement in tumor progression and immunity, paving the way for new biomarkers to improve diagnostics and treatment efficacy and safety (6, 7).

Integrins, which include 18 α and 8 β subunits forming 24 different heterodimeric transmembrane receptors, are crucial in the regulation of cellular signaling and various biological functions such as growth, survival, differentiation, migration, and apoptosis (8). The elevated expression of specific integrins, including αvβ3, αvβ5, α5β1, α6β4, α4β1, and αvβ6, has been linked to the progression of various cancers (9). Therapeutic agents targeting integrins, critical in cancer, are currently undergoing clinical evaluations (9, 10). However, despite the promising antitumor effects, integrin inhibitors fail to significantly improve 5-year survival rates, highlighting the urgent need to explore the detailed molecular mechanisms of integrins and develop new, effective, low-toxicity inhibitors (11).

ITGA4 is one of the less studied integrin family members (12), playing a crucial role in mediating cell-cell adhesions that are vital for immune functionality (13). ITGA4 forms two key integrin complexes by pairing with β1 (CD29) to create α4β1 (very late antigen-4, VLA-4) and with β7 to form α4β7 (lymphocyte Peyer patch adhesion molecule). These complexes play roles in immune surveillance, inflammation, and the pathogenesis of cardiovascular disorders (14). ITGA4 is targeted in therapies for multiple sclerosis (MS), Crohn’s disease, and inflammatory bowel disease (IBD) (15). Moreover, it is considered a potential therapeutic target in cancer treatments, affecting tumor development (16, 17). However, ITGA4’s specific functions in cancer progression and the tumor microenvironment (TME) remain elusive. Current research is often limited to single cancer types, potentially missing broader insights. To address this gap, our study conducted a comprehensive pan-cancer analysis using multi-omics data to evaluate ITGA4’s diagnostic and prognostic capabilities, with a nomogram based on ITGA4 expression was developed to assist. We also performed functional enrichment analysis to uncover molecular mechanisms involving ITGA4. Additionally, we investigated ITGA4’s influence on tumor immunity, heterogeneity, stemness, drug sensitivity. Validation of our findings involved analyzing single-cell and transcriptome sequencing data of GC, accompanied by *in vitro* experiments and clinical data analysis. These efforts demonstrate ITGA4’s potential as a significant biomarker in oncology, highlighting its potential role in tumor immunotherapy.

## 2 Materials and methods

### 2.1 Analysis of ITGA4 expression in pan-cancer and subgroups

We investigated ITGA4 expression at the single-cell transcriptomic level in healthy infants and adults using the Human Transcriptome Cell Atlas (HTCA) database (https://www.htcatlas.org/) (18). For examining the expression differences of ITGA4 between pan-cancer and normal tissues, we obtained pan-cancer datasets from the Xena database (https://xenabrowser.net/) (19), which include transcriptome sequencing data (TPM format) and clinical information from The Cancer Genome Atlas (TCGA, https://portal.gdc.cancer.gov/) and the Gene Type-Tissue Expression (GTEx, https://www.gtexportal.org/) (20) projects. Cancer types with fewer than three samples were excluded, and gene expression data were standardized using log_2_(x + 0.001). Data processing and visualization were performed using R packages “AnnotationDbi”, “org.Hs.eg.db”, “stringr”, “stringi”, “ggplot2”, and “RColorBrewer”. Additionally, the “CPTAC” section of the UALCAN database (https://ualcan.path.uab.edu/index.html) (21) was employed to analyze the ITGA4 protein levels. The correlation between ITGA4 expression and tumor grade and stage was analyzed using the “limma” and “stringr” packages. Finally, the TISIDB database (http://cis.hku.hk/TISIDB/) (22) was used to analyze ITGA4 expression patterns across different molecular and immune subtypes.

### 2.2 Evaluation of the diagnostic and prognostic value of ITGA4

To assess ITGA4’s diagnostic value, we utilized the R package “pROC” to plot receiver operating characteristic (ROC) curves. An area under the curve (AUC) above 0.7 indicated diagnostic value, above 0.8 indicated good accuracy, and above 0.9 indicated excellent diagnostic value. Subsequently, the “survival” and “forestplot” packages were used to generate forest plots for Cox proportional hazards regression models (23). Kaplan-Meier (KM) curves for overall survival (OS), disease-specific survival (DSS), disease-free interval (DFI), and progression-free interval (PFI) were plotted using the “survminer” and “ggplot2” packages. High and low ITGA4 expression groups were determined based on the median ITGA4 expression value. We then used the “timeROC” and “ggplot2” packages to create time-dependent ROC curves, evaluating ITGA4’s ability to predict 1-, 3-, and 5-year survival rates. We randomly selected 70% of the TCGA pan-cancer samples as a training set (6830/9784) and used the “rms” and “survival” packages to construct a nomogram model for predicting patient prognosis. Model accuracy was verified with calibration plots. Finally, we evaluated the model’s clinical decision-making value and predictive accuracy for 1-, 3-, and 5-year survival rates using time-dependent ROC curves with the “timeROC” package and decision curve analysis (DCA) with the “ggDCA” package for both the training set and validation set (2954/9784).

### 2.3 Analysis of ITGA4 interaction proteins and functional enrichment

We constructed protein-protein interaction (PPI) networks for ITGA4 using the GeneMANIA (http://www.genemania.org) (24), STRING (https://cn.string-db.org/) (25), and Cytoscape software. Gene ontology (GO) and Kyoto Encyclopedia of Genes and Genomes (KEGG) pathway enrichment analyses for these proteins were performed and visualized using the R packages “clusterProfiler”, “org.Hs.eg.db”, “tidyr”, “ggplot2”, and cnetplot function (26). Additionally, we explored the potential functions of ITGA4 at the single-cell level using the CancerSEA database (http://biocc.hrbmu.edu.cn/CancerSEA/) (27). We identified hub proteins by intersecting the top 70 ITGA4-associated proteins from both databases. The results were visualized with Venn diagrams using “VennDiagram” and “ggplot2” packages and a correlation heatmap of ITGA4 and hub gene expression was created using “ComplexHeatmap” package (28). Functional enrichment analysis of the hub genes was subsequently performed using the GSCA database (https://guolab.wchscu.cn/GSCA/#/) (29).

### 2.4 Correlation analysis of ITGA4 and cancer immunity

We analyzed the Spearman correlation between ITGA4 and three TME scores (StromalScore, ImmuneScore, and ESTIMATEScore) using the R package “ESTIMATE”. Spearman correlation analysis between ITGA4 expression and immune cell infiltration was assessed using five algorithms—CIBERSORT, xCELL, TIMER, EPIC, and MCPCounter—via the “IOBR” and “psych” package. Correlation heatmaps were generated using “ggplot2” to visualize these results. We also used the TISIDB database (http://cis.hku.hk/TISIDB/index.php) (30) to examine the relationships between ITGA4 and various tumor-infiltrating lymphocytes (TILs), immunoregulatory genes (immunostimulators, immunoinhibitors, and major histocompatibility complex (MHC) genes), chemokines, and chemokine receptors across cancers (31, 32).

### 2.5 Correlation analysis of ITGA4 with MMR, tumor heterogeneity, stemness, and immunotherapy responsiveness

We visualized the correlation between ITGA4 and four key MMR genes (MLH1, MSH2, MSH6, and PMS2) using heatmaps generated with the “ggplot2” package. Utilizing the “TMB” and “inferHeterogeneity” functions in the “maftools” package, and referencing previous studies (33, 34), we obtained various tumor heterogeneity parameters, including TMB, mutational and clonal intratumoral heterogeneity (MATH), MSI, neoantigen load (NEO), tumor purity, ploidy, homologous recombination deficiency (HRD), and loss of heterozygosity (LOH). Additionally, we sourced tumor stemness scores such as RNAss, EREG.EXPss, DNAss, DMPss, ENHss, and EREG-METHss from existing research (35). “ggplot2” was used to create correlation heatmaps. Furthermore, lollipop plots illustrating TMB and MSI’s correlations with ITGA4 were generated with “ggplot2” package. To further explore the predictive role of ITGA4 and other biomarkers on immune checkpoint blockade (ICB) therapy responsiveness, we employed the “Biomarker Evaluation” and “Regulator Prioritization” modules of the Tumor Immune Dysfunction and Exclusion (TIDE) database (http://tide.dfci.harvard.edu). We also analyzed ITGA4 expression in various immunosuppressive datasets. Finally, we assessed ITGA4 expression changes pre- and post-ICB therapy in tumor models and pre- and post-cytokine therapy in tumor cell lines using the TIMSO database (http://tismo.cistrome.org/) (36).

### 2.6 Mutation and methylation analysis of ITGA4 in pan-cancer

We used the cBioPortal database (https://www.cbioportal.org/) (37) to investigate ITGA4 mutation frequency, types, distribution, specific sites, and the relationship between ITGA4 expression and copy number alterations (CNA). We then examined the top ten genes most likely to mutate in the ITGA4-mutant group compared to the non-mutant group. The SMART database (http://www.bioinfo-zs.com/smartapp/) (38) was used to analysis ITGA4 methylation levels and its 18 specific methylation sites, as well as the correlations between them in cancer and normal tissues.

### 2.7 ITGA4 expression and drug sensitivity correlation analysis

We obtained therapeutic sensitivity data for anti-cancer drugs from CellMiner (https://discover.nci.nih.gov/cellminer/) (39). Using the R packages “impute,” “limma,” “ggplot2,” and “ggpubr,” we analyzed and plotted the correlation between drug sensitivity and ITGA4 expression levels. The 3D structures of the ITGA4 protein and chemotherapeutic drugs were downloaded from the AlphaFold Protein Structure Database (https://www.alphafold.ebi.ac.uk/) (40, 41) and the PubChem platform (https://pubchem.ncbi.nlm.nih.gov/), respectively. Molecular docking and visualization of the results were performed using AutoDockTool (version 1.5.7) and the Pamon (version 3.0.3).

### 2.8 Prognostic impact, functions, immune correlation, and single-cell analysis of ITGA4 in GC

Transcriptome data (TPM format) and prognostic information for the TCGA-STAD cohort were obtained from the Xena database. KM curves were plotted to compare the OS differences between patients with the highest 20% and lowest 20% ITGA4 expression levels, following the method outlined in the above sections. Patients were divided into high and low ITGA4 expression groups based on the median expression level. Differentially expressed genes (DEGs) between these groups were identified using “DESeq2,” “edgeR,” and “ggplot2” packages (*|log_2_FC|* > 1, adjusted *P* < 0.05) and visualized with a volcano plot (42, 43). GO and KEGG enrichment analyses of these DEGs were also performed using the method described in the above sections. Cancer reference gene sets (c2.cp.kegg.v2022.1.Hs.symbols.gmt) were obtained from the Molecular Signatures Database (MsigDB, http://www.gsea-msigdb.org/gsea/). After ID conversion using the “org.Hs.eg.db” package, DEGs were ranked by log2FC, and Gene Set Enrichment Analysis (GSEA) (44) was conducted using the “clusterProfiler” package, with results visualized by “ggplot2.” We also analyzed the correlation between ITGA4 expression and three TME scores, as well as immune cell infiltration in GC, following the method from the “Correlation analysis of ITGA4 and cancer immunity” section. The single-cell RNA sequencing data and annotation files from the GSE134520 and GSE167297 datasets were obtained from the Tumor Immune Single-cell Hub (TISCH, http://tisch.comp-genomics.org/home/) (45) and were analyzed using the “MAESTRO” and “Seurat” packages with t-SNE for cell clustering.

### 2.9 Cell culture

Human GC cell lines MKN45, HGC27, AGS, KATO III, N87, and gastric mucosal epithelial cell line GES-1, authenticated by short tandem repeat (STR) analysis, were obtained from the Digestive System Tumor Prevention and Treatment and Translational Medicine Engineering lnnovation Center of Lanzhou University. The cells were cultured in RPMI-1640 medium (Cat: 11875101, *Gibco*) containing 10% fetal bovine serum (FBS, Cat: G8002, *Servicebio*) and 1% penicillin/streptomycin (Cat:C0222, *Beyotime*) at 37°C in a humidified incubator (POG-150, *BOLV INSTRUMENT*) with 5% CO_2_.

### 2.10 Western blot

Protein levels of ITGA4 were analyzed in six pairs of GC and adjacent tissues collected from gastrectomy patients at the Second Hospital of Lanzhou University (January-December 2023, with ethical approval from the Ethics Committee of the Second Hospital of Lanzhou University and informed consent from patients), and in the six cell lines mentioned earlier. The staging of all six GC clinical samples was classified as stage III. Tissues and cells were lysed on ice using a lysis buffer with a PSMF (Cat: P0100, *Solarbio*):RIPA (Cat: P0013B, *Beyotime*) ratio of 1:100 to extract proteins. Equal amounts of protein samples were separated using 10% SDS-PAGE gels and transferred to PVDF membranes (Cat: IPVH00010, *Millipore*). Membranes were blocked with 5% skim milk (Cat: D8340, *Solarbio*) for 1 hour, then incubated overnight at 4°C with primary antibodies against ITGA4 (1:1000, Cat: 19676-1-AP, *Proteintech*) and GAPDH (1:10000, Cat: 60004-1-Ig, *Proteintech*). Afterward, membranes were incubated with HRP-conjugated secondary antibodies (1:10000, Cat: SA00001-2, *Proteintech*) for 1 hour at room temperature. Protein bands were visualized using an enhanced chemiluminescence (ECL) imaging system (JP-600Plus, *JIAPENG*) and quantification was performed using ImageJ software.

### 2.11 siRNA transfection

Cells were seeded into a six-well plate (2×10^5^ cells/well). After attachment, cells were transfected with siRNA (20 μM) and Lipo6000 (Cat: C0526-0.5ml, *Beyotime*) following the manufacturer’s protocol (*GENERAL BIO*). Specifically, 3 μL siRNA and 5 μL Lipo6000 were diluted in 250 μL Opti-MEM (Cat: 31985062, *Thermo*) each, mixed, and added to 1500 μL 1640 complete medium. The transfection mixture was applied to the cells and incubated at 37°C with 5% CO_2_ for 6 hours before replacing with fresh 1640 medium. ITGA4 expression was analyzed by WB 48 hours post-transfection. The siRNA primer sequences for ITGA4 (human) were: 5’-CGAACAGAACUGAGUAAAA(dT)(dT)-3’ and 5’-CCUACAACGUGGACACUGA(dT)(dT)-3’.

### 2.12 Wound-healing assay

Cells were cultured in 6-well plates with RPMI-1640 medium (10% FBS) until confluent. A sterile scraper created a scratch, washed with PBS (Cat: F211131, *BasalMedia*), and incubated at 37°C, 5% CO2. Scratch width was imaged at 0 and 24 hours and analyzed with ImageJ.

### 2.13 Flow cytometry analysis

Cells were washed with cold PBS 3 times, resuspended in 100 µL binding buffer, and incubated with 5 µL AV/APC or 7-AAD solution for 15 minutes in the dark (Cat: AP105, *Multi Sciences)*. After adding 400 µL binding buffer, apoptosis rate was assessed by flow cytometry (*BD Biosciences*).

### 2.14 ITGA4 association with clinicopathological features and serum markers in GC patients via immunohistochemistry

With approval from the Ethics Committee of the Second Hospital of Lanzhou University, we collected 80 pairs of paraffin-embedded GC and adjacent tissue samples from June 2020 to December 2023. After sectioning, samples were deparaffinized, rehydrated, and immersed in antigen retrieval solution (Cat: 005000, *Thermo*) under high pressure (150-200 kPa) for 10 minutes. The sections were then incubated with 3% hydrogen peroxide (Cat: 88597, *Merck*) for 20 minutes, followed with 10% BSA (Cat: 37520, *Thermo*) at room temperature for 60 minutes. Next, the sections were incubated with primary anti-ITGA4 antibody (1:100, Cat: 19676-1-AP, *Proteintech*) overnight at 4°C, then with secondary antibody (1:2000, Cat: A-11008, *Thermo*) for 1 hour. Thereafter, DAB staining (Cat: PR30010, *Proteintech*) and hematoxylin counterstaining (Cat: PR30004, *Proteintech*) were performed. ITGA4 expression was quantified using a digital imaging system (*3DHISTECH*, *Hungary*). Staining intensity was scored from 0 to 3 (0 for no staining, 1 for pale yellow, 2 for light brown, and 3 for dark brown), and the percentage of positive cells was divided into four equal grades from 0-100%, corresponding to scores from 1 to 4 in ascending order. The final score was the product of the intensity and positive area scores.

We analyzed the correlation between ITGA4 expression and various clinicopathological features, as well as serum tumor and immune markers, in the previously mentioned 80 GC cases. These entries included prognostic and diagnostic value, gender, age, TNM stage, pathological stage, perineural invasion, vascular invasion, gastric mucosal ulceration/bleeding, and serum tumor markers and immune cell levels (detailed in the “Results” section).

### 2.15 Statistical methods

Statistical analysis and visualization were conducted using R software (version 4.2.3). Spearman correlation analysis was utilized to evaluate correlations. Results were based in triplicate experiments and were presented as means ± standard deviation (SD). Statistical significance was assessed using log-rank test, student’s *t*-test, Mann–Whitney test, Welch’s *t* test, Wilcoxon test, Chisq test, Yates’ correction and two-way ANOVA. *P*-values less than 0.05 considered statistically significant.

## 3 Results

### 3.1 ITGA4 expression in pan-cancer contexts

**Figure 1:**
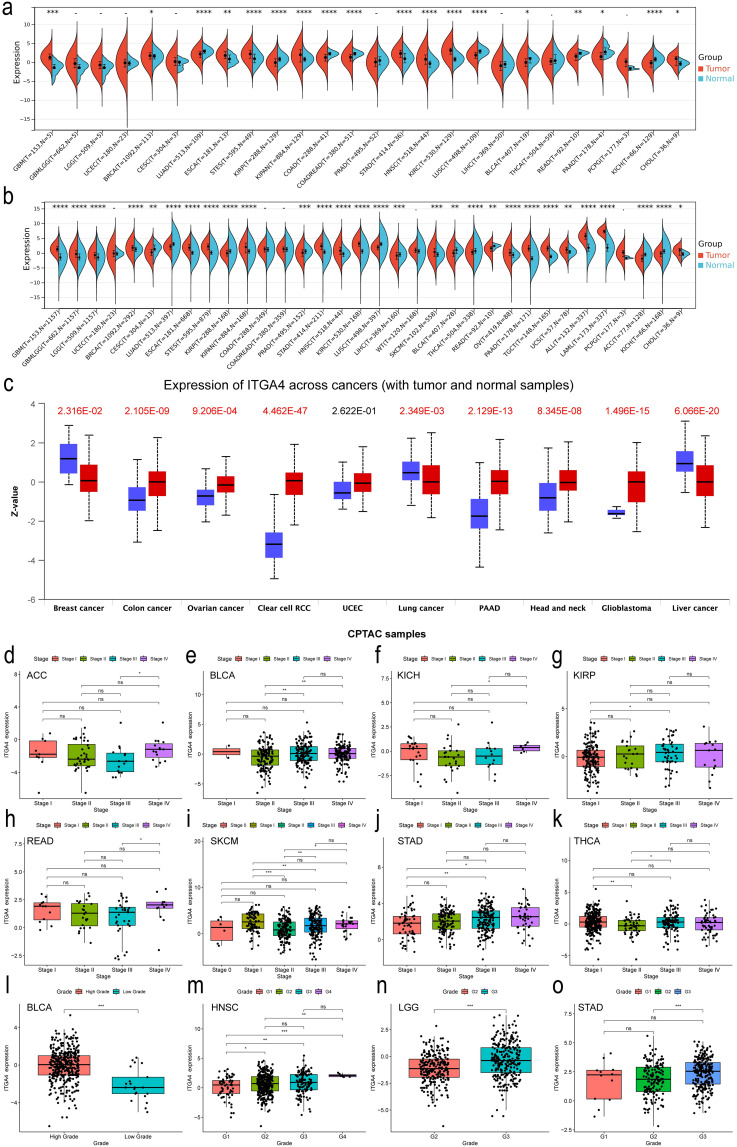
Expression and clinical relevance of ITGA4 in pan-cancer. **(A, B)** Expression of ITGA4 in tumor and normal tissues from the TCGA database and the TCGA+GTEX database, respectively. **(C)** Protein levels of ITGA4 between cancerous and normal tissues from the CPTAC database. **(D–K)** ITGA4 expression across different tumor stages in ACC, BLCA, KICH, KIRP, READ, SKCM, STAD, and THCA. **(L–O)** ITGA4 expression across different tumor grades in BLCA, HNSC, LGG, and STAD. (-/*ns*, no significance; *P < 0.05; **P < 0.01; ***P < 0.001; ****P < 0.0001).

We first examined the expression of ITGA4 in normal tissues. HTCA database analysis showed low ITGA4 expression in most adult and infant tissues (**Supplementary Figure S1A**), indicating its potential as a therapeutic target with low tissue toxicity. We then analyzed ITGA4 mRNA levels using the TCGA database, observing significant expression differences in most cancer types (18 of 24) (**Figure 1A**). Further analysis combining TCGA and GTEx databases confirmed these variations in 29 of 34 cancer types (**Figure 1B**). Notably, cancers like GBM, BRCA, ESCA, STES, KIPAN, STAD, HNSC, KIRC, and CHOL consistently showed higher ITGA4 expression in individual and combined databases, while LUAD, KIRP, LUSC, BLCA, READ, and KICH consistently exhibited lower expression. Additionally, analysis from the CPTAC database revealed that ITGA4 protein expression was higher in tumor tissues compared to normal tissues in COAD, OV, KIRC, PAAD, HNSC, and GBM, while it was lower in LIHC, LUAD, and BRCA (**Figure 1C**).

Our results further demonstrated that ITGA4 levels were elevated in higher stages compared to lower stages in ACC, BLCA, KICH, KIRP, READ, and STAD, whereas a reverse trend was observed in SKCM and THCA (**Figures 1D–K**). Moreover, higher grades of BLCA, HNSC, LGG, and STAD displayed increased ITGA4 expression compared to their lower counterparts, with notable elevations in both higher grades and stages for STAD and BLCA (**Figures 1L–O**). Using the TISIDB database, we explored potential correlations between ITGA4 expression and the molecular and immune subtypes of various cancers, noting variable ITGA4 expression in molecular subtypes within BRCA, HNSC, KIRP, LGG, LIHC, OV, PCPG, STAD, and UCEC—for example, elevated expression levels in STAD’s EBV subtype and HNSC’s Mesenchymal subtype (**Supplementary Figure S1B**). Concurrently, ITGA4 expression varied across different immune subtypes in 23 cancer types (**Supplementary Figure S1C**). These observations underscore the complex expression patterns of ITGA4 in tumors, suggesting its significant role in tumor progression and potential utility in tailoring clinical strategies for different cancer subtypes.

### 3.2 Diagnostic and prognostic efficacy of ITGA4 in pan-cancer

We evaluated the diagnostic value of ITGA4 in distinguishing tumor from normal tissues using ROC curves. ITGA4 demonstrated diagnostic potential in 20 cancer types (AUC>0.7). Specifically, PAAD, SARC, and TGCT showed AUC values exceeding 0.9, with LAML, whose AUC is 1, demonstrating perfect diagnostic accuracy (**Supplementary Figures S2A–C**).

**Figure 2:**
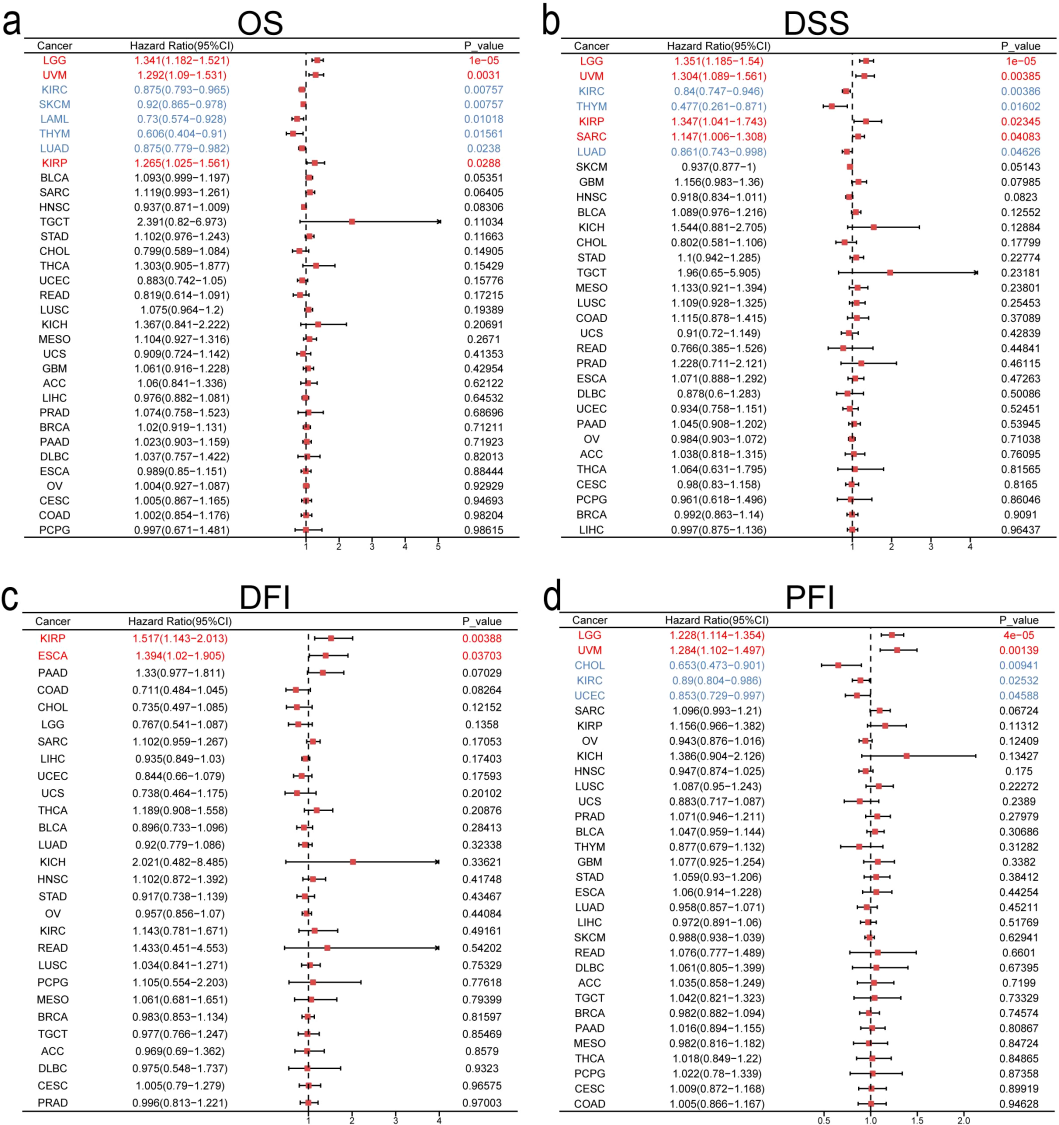
Univariate COX regression analysis of ITGA4 in pan-cancer for OS **(A)**, DSS **(B)**, DFI **(C)**, and PFI **(D)**.

Subsequently, using univariate Cox regression forest plots and KM survival curves, we assessed the prognostic implications of ITGA4 expression across different cancers. Forest plots for OS revealed that ITGA4 is a risk factor in KIRP, LGG, and UVM, but acts as a protective factor in KIRC, LAML, LUAD, SKCM, and THYM (**Figure 2A**). These results were validated by KM survival curves, which demonstrated that high ITGA4 expression was associated with lower OS in LGG and UVM, while it correlated with higher survival probabilities in KIRC, LUAD, SKCM, and HNSC (**Supplementary Figure S3A**). Further exploration showed that elevated ITGA4 expression could negatively influence DSS in KIRP, LGG, SARC, and UVM, but it lowered the risk of adverse DSS outcomes in KIRC, LUAD, and THYM (**Figure 2B**). KM curves for DSS in KIRC, UVM, LGG, and LUAD corroborate these findings, and high ITGA4 expression also correlated with poorer DSS in KICH and UCEC, whereas it prolonged DSS in HNSC (**Supplementary Figure S3B**). In terms of DFI, high ITGA4 expression significantly correlated with shorter DFI in ESCA and KIRP (**Figure 2C**). KM analysis further indicated that ITGA4 might serve as a prolonging factor for DFI in CHOL (**Supplementary Figure S3C**). PFI analysis showed that ITGA4 as a protective factor in CHOL, KIRC, and UCEC, but demonstrated an adverse effect in LGG and UVM (**Figure 2D**), a finding supported by KM analysis (**Supplementary Figure S3D**).

**Figure 3:**
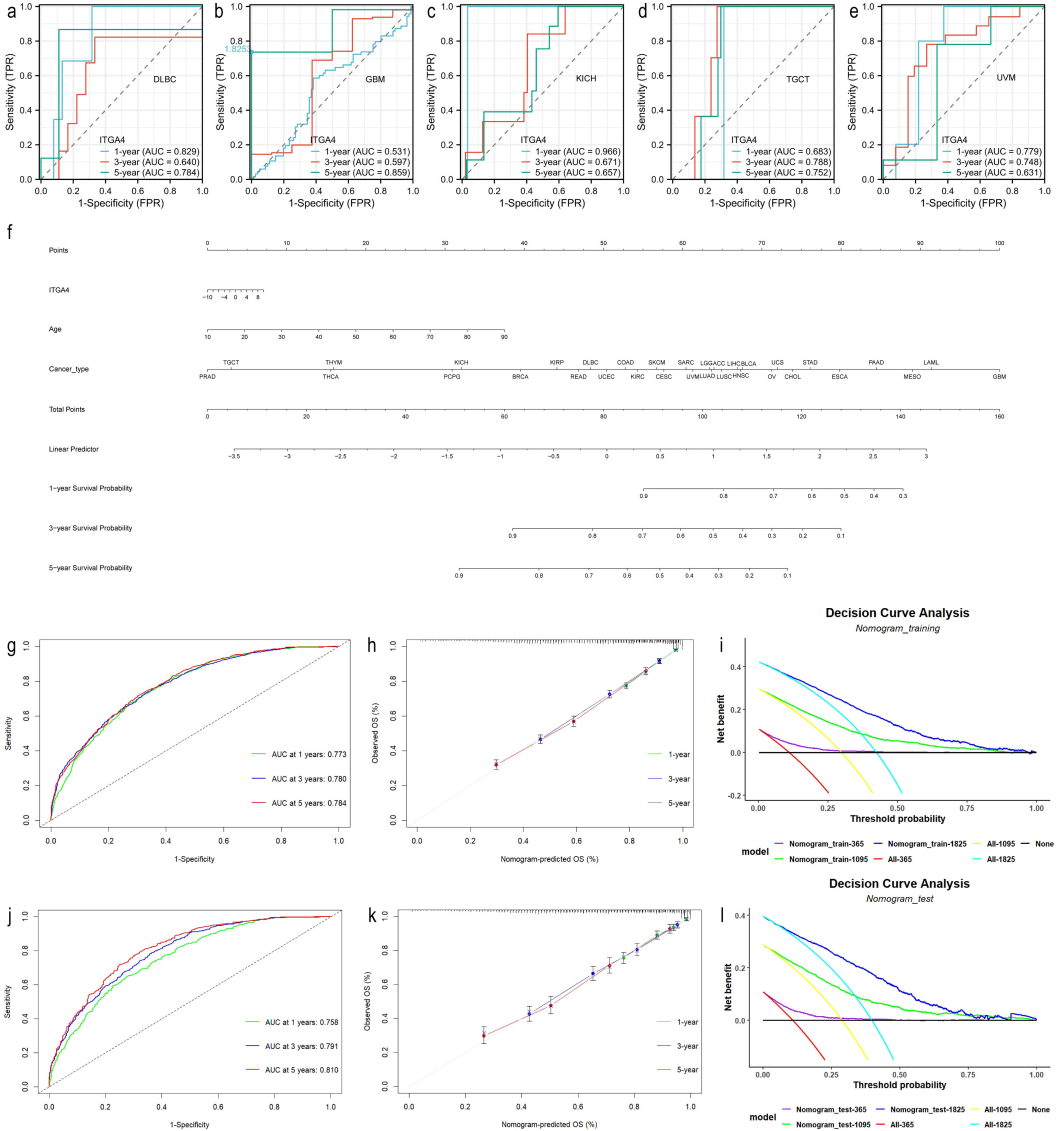
Predictive and clinical decision-making value of ITGA4. **(A–E)** Time-dependent ROCs for 1-, 3-, and 5-year survival predictions of DLBC, GBM, KICH, TGCT, and UVM. **(F)** Nomogram predicting 1-, 3-, and 5-year survival rates for various cancer patients, established based on age, ITGA4 expression, and tumor type. **(G–I)** Time-dependent ROC, calibration curves, and DCA of the predictive model in the training set (6830/9784). **(J–L)** Time-dependent ROC, calibration curves, and DCA of the predictive model in the validation set (2954/9784).

To evaluate ITGA4’s predictive value for 1-, 3-, and 5-year survival, we produced time-dependent ROC curves. ITGA4 demonstrated strong predictive capabilities, such as an AUC of 0.784 for 5-year DLBC survival, 0.966 for 1-year KICH survival, and over 0.7 for both 3-year and 5-year TGCT survival (**Figures 3A–E**). We then developed a nomogram using TCGA data based on ITGA4 expression, patient age, and cancer type to predict survival probabilities (**Figure 3F**), which showed strong predictive performance in both training and validation datasets with AUC exceeding 0.7 for 1-, 3-, and 5-year survival (**Figures 3G, J**), validated by accurate calibration curves (**Figures 3H, K**). DCA on both training and testing datasets showed that models like All-1825 and All-1095 provided higher net benefits at low to medium thresholds, demonstrating their predictive value. Conversely, the All-365 model displayed limited utility at higher thresholds (**Figures 3I, L**). These findings emphasize the importance of ITGA4 expression-model selection based on clinical context to optimize patient outcomes, underscoring the value of tailored clinical decision-making.

### 3.3 Functional analysis of ITGA4 in cancer

**Figure 4:**
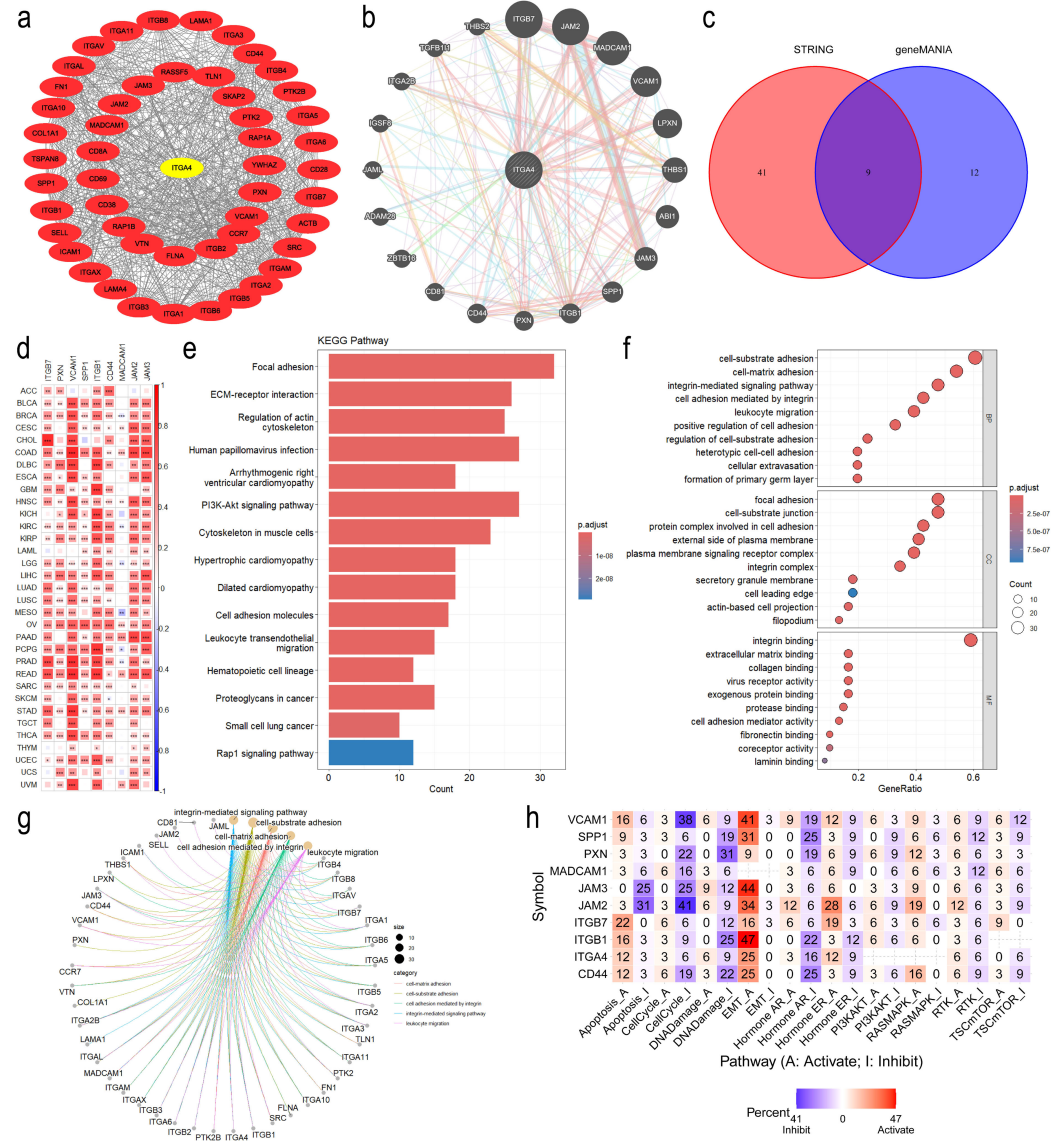
Functional enrichment analysis of ITGA4. **(A)** PPI network of ITGA4 and its 50 most related genes from the STRING database. **(B)** PPI network of ITGA4 and its 20 most related genes from the geneMANIA database. **(C)** Venn diagram identifying nine hub genes. **(D)** Heatmap depicting the correlation between ITGA4 and the nine hub genes. **(E)** KEGG pathway enrichment analysis. **(F)** GO enrichment analysis. **(G)** Visualization of GO enrichment analysis results using the cnetplot function. **(H)** Heatmap of functional enrichment analysis of ITGA4 and the nine hub genes based on the GSCA database. (*P < 0.05; **P < 0.01; ***P < 0.001).

We used STRING and geneMANIA databases to identify the top 20 and 50 proteins associated with ITGA4 respectively, with PPI networks constructed (**Figures 4A, B**). The intersection of these datasets revealed nine hub genes, with a pan-cancer heatmap confirming their positive correlation with ITGA4 (**Figures 4C, D**). Functional enrichment analysis of these 70 genes identified KEGG categories like ECM-receptor interaction, the PI3K-Akt signaling pathway, cell adhesion molecules, leukocyte transendothelial migration, and the Rap1 signaling pathway (**Figure 4E**). Correspondingly, GO enrichment analysis highlighted the involvement of ITGA4 in cell-substrate adhesion, integrin-mediated cell adhesion, extracellular matrix binding, leukocyte migration, and cellular extravasation (**Figures 4F, G**). Data from the CancerSEA database showed at the single-cell level that ITGA4 may impact cellular quiescence, differentiation, apoptosis, and processes related to cancer metastasis, invasion, and angiogenesis (**Supplementary Figures S4A–F**). To augment our comprehension of ITGA4’s roles in tumor progression, analyses of the aforementioned nine hub genes via the GSCA database indicated their potential roles in regulating apoptosis, the cell cycle, DNA damage repair, epithelial-mesenchymal transition (EMT), and key oncogenic signaling pathways including PI3K-AKT, RAS-MAPK, RTK, and TSC-mTOR pathways (**Figure 4H**). These analyses deepen our understanding of ITGA4’s role in tumor biology and microenvironments, offering crucial insights for developing targeted therapies and predicting tumor behaviors.

### 3.4 ITGA4’s role in TME, immune infiltration and immunomodulators

**Figure 5:**
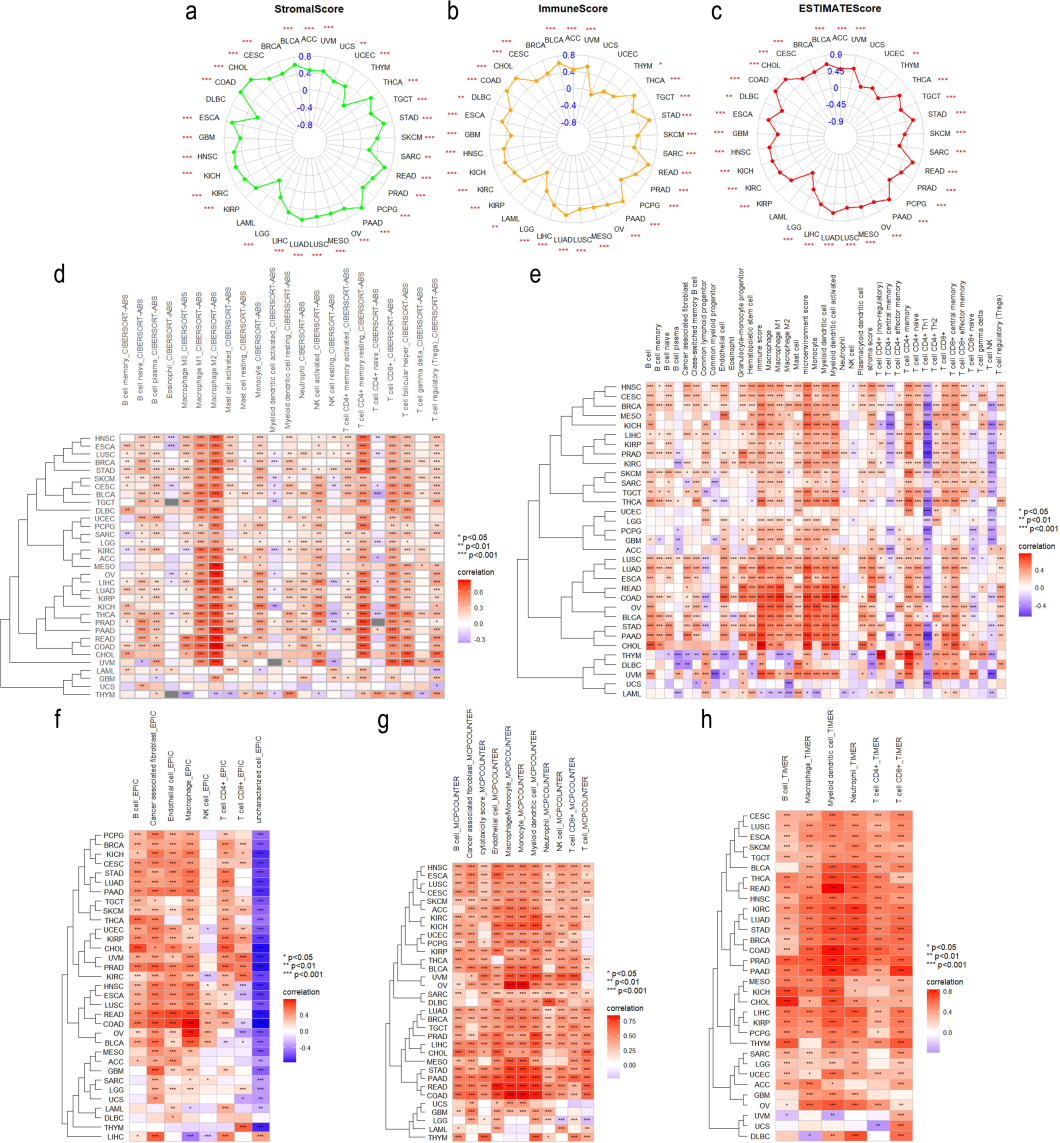
Tumor Immunology correlation of ITGA4. Radar plots illustrating the correlation of ITGA4 with StromalScore **(A)**, ImmuneScore **(B)**, and ESTIMATEScore **(C)** across various cancers. Heatmaps depicting the correlation between ITGA4 and tumor immune cell infiltration based on five different algorithms: CIBERSORT **(D)**, xCELL **(E)**, EPIC **(F)**, MCPCounter **(G)**, and TIMER **(H)**. (*P < 0.05; **P < 0.01; ***P < 0.001).

As TME has an important impact on tumor activity and response to treatment (46), we analyzed the correlation between ITGA4 expression and TME scores, revealing significant positive correlations with StromalScore, ImmuneScore, and ESTIMATEScore (**Figures 5A–C**). These findings suggest that tumors with high ITGA4 expression may have a rich stromal component, extensive immune cell infiltration, and high tumor purity. This indicates a potential pivotal role of ITGA4 in modulating the TME, particularly in the interactions between tumor cells and the surrounding stroma and immune cells.

We then conducted a detailed analysis using five distinct algorithms to elucidate the relationship between ITGA4 expression and immune cell infiltration across various cancer types. Overall, except for UCS, ITGA4 expression showed significant correlations with the infiltration of multiple immune cell types, especially macrophages, CD8^+^ T cells, and CD4^+^ T cells, in most cancers (**Figures 5D–H**). Specifically, results from the CIBERSORT algorithm revealed significant positive correlations between ITGA4 and both M1 and M2 macrophage types, which are antagonistic. Notably, the association with M2 macrophages generally exceeded that with M1 macrophages. This pattern could potentially be explained by the significant negative correlation of CD4^+^ Th1 cells with ITGA4 across multiple cancers using the XCELL algorithm (whereas Th2 cells, which are antagonistic to Th1 cells, showed significant positive correlations with ITGA4), since a decrease in Th1 cell infiltration could diminish the activation of M1 macrophages through reduced interferon-gamma (IFN-γ) secretion. We hypothesize that abnormal ITGA4 expression may foster an adverse inflammatory environment in tumors, leading to decreased M1 macrophage activation while facilitating their polarization towards the tumor-promoting M2 phenotype. The recruitment of CD4^+^ and CD8^+^ T cells to tumor sites is likely influenced by this inflammatory milieu and regulated by ITGA4. Furthermore, regulatory T cells (Tregs), cancer-associated fibroblasts (CAFs), and endothelial cells, also demonstrated significant positive correlations with ITGA4 in multiple cancers. We further analyzed the association of ITGA4 with TILs, immunoregulators, chemokines, and their receptors using the TISIDB database. Consistent with prior results, ITGA4 significantly correlated with many immune cells across different tumors (**Supplementary Figure S5A**). Moreover, ITGA4 exhibited significant associations with a broad range of Immune regulatory genes, including immunostimulators, immunoinhibitors, and MHC molecules (**Supplementary Figure S5B–D**). Additionally, ITGA4 showed substantial positive correlations with several chemokines and their receptors, notably including multiple members of the CCL, CXCL, CCR, and CXCR gene families (**Supplementary Figure S5E, F**). It’s worth noting that TME score, immune cell infiltration, and immune regulation all showed weak or inconsistent correlations with ITGA4 expression in LAML and UCS, warranting further investigation. The above findings underscore the complex role and potential importance of ITGA4 in regulating the tumor immune microenvironment, providing a theoretical foundation for future therapies targeting ITGA4.

### 3.5 Correlations between ITGA4 and MMR, tumor heterogeneity, stemness and immunotherapy response

**Figure 6:**
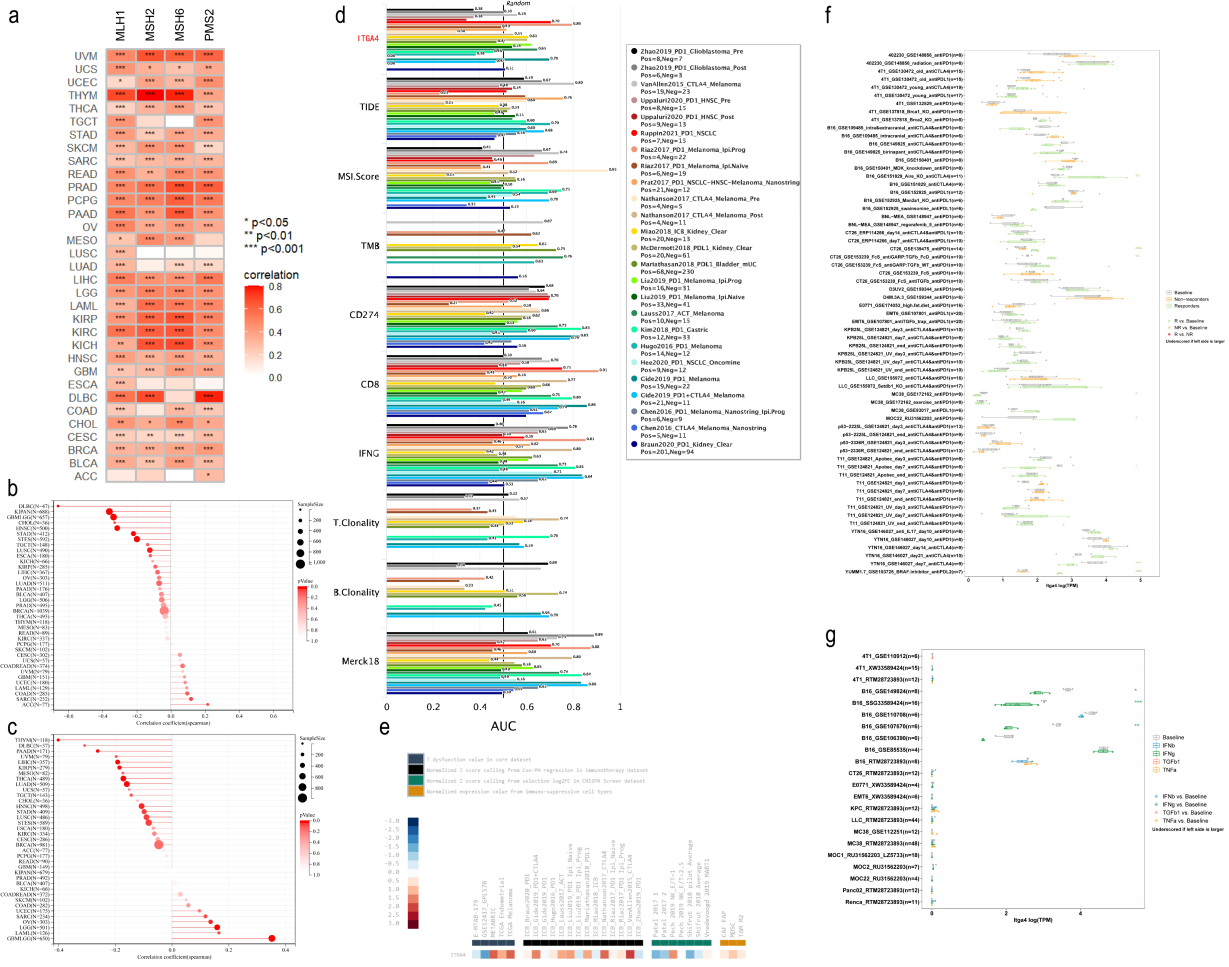
Correlation of ITGA4 with MMR, MSI, TMB, and immune therapy response. **(A)** Heatmap of the correlation between ITGA4 and four key MMR genes. Lollipop plots depicting the correlation of ITGA4 with MSI **(B)** and TMB **(C)**. **(D)** The predictive value of ITGA4 and other biomarkers for ICB therapy responsiveness. **(E)** Expression of ITGA4 in immunosuppressive and immunotherapy cohorts. **(F)** Correlation of ITGA4 with *in vivo* tumor model response to immunotherapy. **(G)** Relationship between ITGA4 and *in vitro* cell line response to cytokine therapy. (*P < 0.05; **P < 0.01; ***P < 0.001).

Given the significant impact of tumor cell heterogeneity and stemness on malignancy and immunotherapy sensitivity, we explored the relationship between ITGA4 and related indices across multiple cancers. We initially investigated the association between ITGA4 and four key MMR genes (MLH1, MSH2, MSH6, PMS2) and found a significant positive correlation in 30 cancer types, excluding ACC, ESCA, and LUSC (**Figure 6A**). Further analysis revealed that ITGA4 was significantly negatively correlated with MSI in seven cancer types and with TMB in ten types (**Figures 6B, C**). This suggests that elevated ITGA4 expression could decrease MSI and TMB mediated by MMR, potentially complicating tumor responsiveness to immunotherapy. Intriguingly, in GBMLGG, LGG, and OV, a positive correlation between ITGA4 and TMB was observed, likely due to unintended mutations in MMR genes. Significant correlations were also noted between ITGA4 and other tumor heterogeneity markers such as HRD, LOH, MATH, NEO, ploidy, and tumor purity across multiple cancer types (**Supplementary Figure S6A**). Moreover, ITGA4 showed a consistent negative correlation with RNAss in most cancers, and its relationship with other stemness scores (EREG.EXPss, DNAss, DMPss, ENHss, EREG-METHss) varied in direction and degree (**Supplementary Figure S6B**), potentially affecting tumor cell drug resistance.

We subsequently evaluated the predictive efficacy of ITGA4 for immunotherapy outcomes in cancer patient cohorts treated with ICB. ITGA4 achieved an AUC over 0.5 in 13 cohorts, comparable to MSI.Score, and outperformed in predicting NSCLC (Ruppin2021_PD1_NSCLC) and melanoma (Riaz2017_PD1_Melanoma_Ipi.Prog, Gide2019_PD1_Melanoma) immunotherapy outcomes (**Figure 6D**). Additionally, ITGA4 expression varied among multiple Immunosuppressive datasets, showing high levels in METABRIC, TCGA Melanoma, ICB_Nathanson2017_CTLA4, and ICB_VanAllen2015_CTLA4 cohorts, but low in GSE12417_GPL570, Patel2017 1, and Shifrut 2018 pilot Average cohorts (**Figure 6E**). Furthermore, analysis using the TIMSO database revealed that ITGA4 could predict immunotherapy response in eight *in vivo* tumor killing assays (primarily involving anti-PD1, anti-CTLA4, and their combination, as well as anti-PDL1 and anti-PDL2) and three *in vitro* cytokine (primarily IFNg) killing assays (**Figures 6F, G**). These findings underscore the potential role and impact of ITGA4 in modulating cancer responses to immunotherapy.

### 3.6 ITGA4 mutations and methylation in pan-cancer

**Figure 7:**
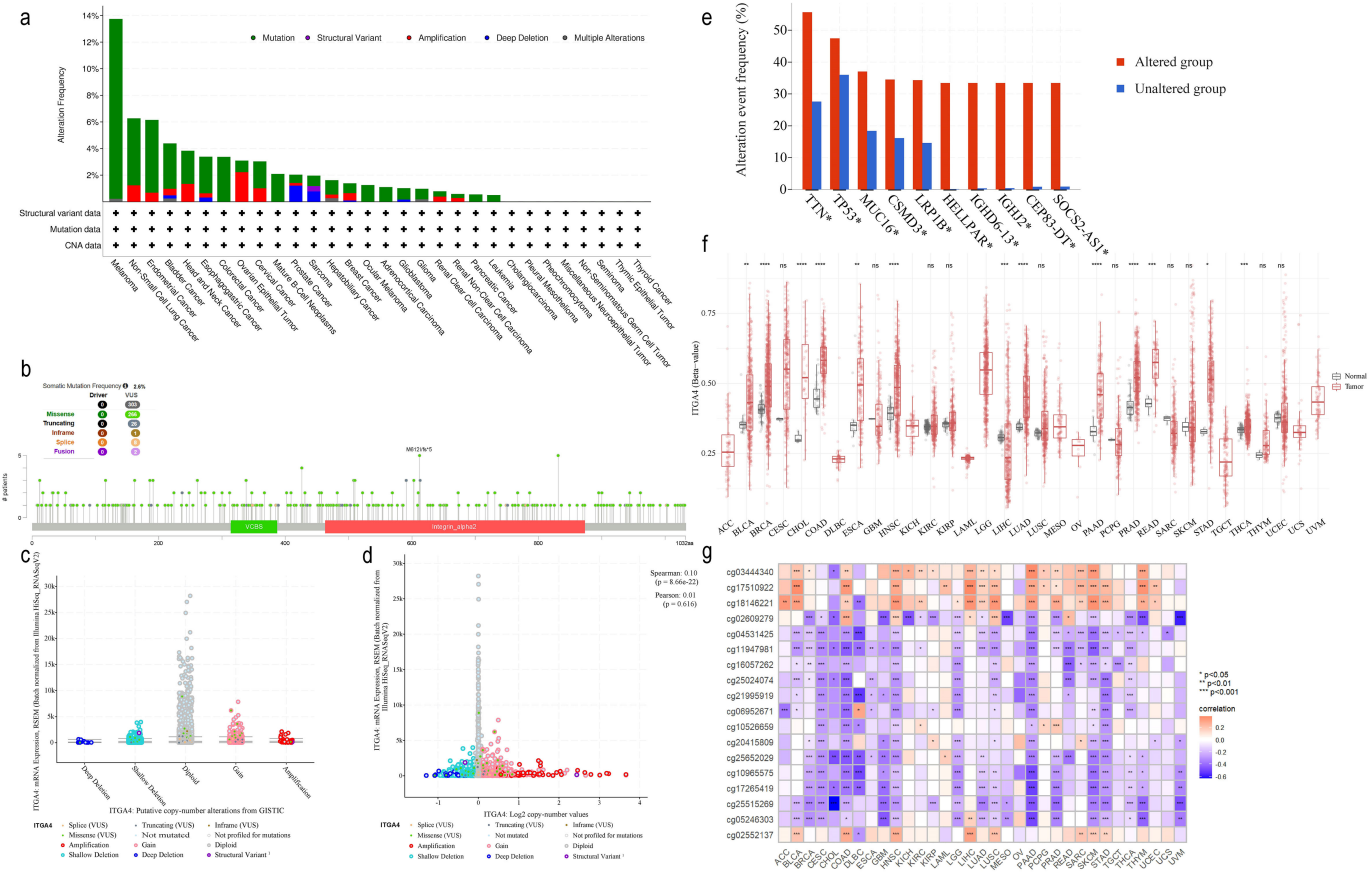
ITGA4 mutation and methylation analysis in pan-cancer. **(A)** The alteration frequency of ITGA4 across various cancer types. **(B)** Mutation sites of ITGA4. **(C)** ITGA4 expression in different CNA categories. **(D)** Scatter plot of the correlation between ITGA4 expression and its copy number values. **(E)** Top ten genes most frequently mutated in the ITGA4 altered group compared to the unaltered group. **(F)** Methylation levels of ITGA4 in pan-cancer versus normal tissues. **(G)** Heatmap depicting the correlation between ITGA4 and its 18 methylation sites. (*ns*, no significance; *P < 0.05; **P < 0.01; ***P < 0.001).

Analysis based on the cBioPortal database revealed ITGA4 alterations in 22 cancer types, with mutations most common, especially in melanoma (**Figure 7A**). Of the 303 somatic mutation sites identified in ITGA4, 266 were missense mutations (**Figure 7B**). **Supplementary Figure S7A** further illustrates the distribution of ITGA4 mutation types across different cancers. Additionally, pan-cancer analysis showed a low correlation between CNAs and ITGA4 expression levels, with no significant differences across CNA types, suggesting minimal impact of CNA on ITGA4 expression in cancer (**Figures 7C, D**). **Figure 7E** displays the top ten genes more prone to mutations in the ITGA4 altered group compared to the non-altered group within the pan-cancer cohort, including TTN, TP53, MUC16, CSMD3, LRP1B, and SOCS2, all known to play roles in tumor progression (47–52).

Methylation plays a critical role in cancer progression (53). Analysis from the SMART database revealed elevated ITGA4 methylation levels in BLCA, BRCA, CHOL, COAD, ESCA, HNSC, LUAD, PAAD, PRAD, READ, STAD, and THCA, but lower levels in LIHC compared to normal tissues (**Figure 7F**). Among the 18 ITGA4 methylation sites analyzed, except for cg05246303 and cg25515269, the remaining 16 sites showed significant variations in over ten types of cancer (**Supplementary Figure S7B**). Furthermore, methylation at cg04531425, cg11947981, cg16057262, cg25024074, cg21995919, cg10965575, cg17265419, cg25515269, and cg05246303 were found to inhibit ITGA4 expression in various cancers. Meanwhile, methylation levels was negatively correlated with ITGA4 expression in CESC, CHOL, ESCA, GBM, MESO, TGCT, THCA, and UVM, and positively correlated in LAML, LIHC, and PCPG (**Figure 7G**). These findings emphasize the potential impact of ITGA4 methylation status on its expression across different cancers, providing insights for new cancer diagnostic or therapeutic strategies.

### 3.7 ITGA4 and drug sensitivity

**Figure 8:**
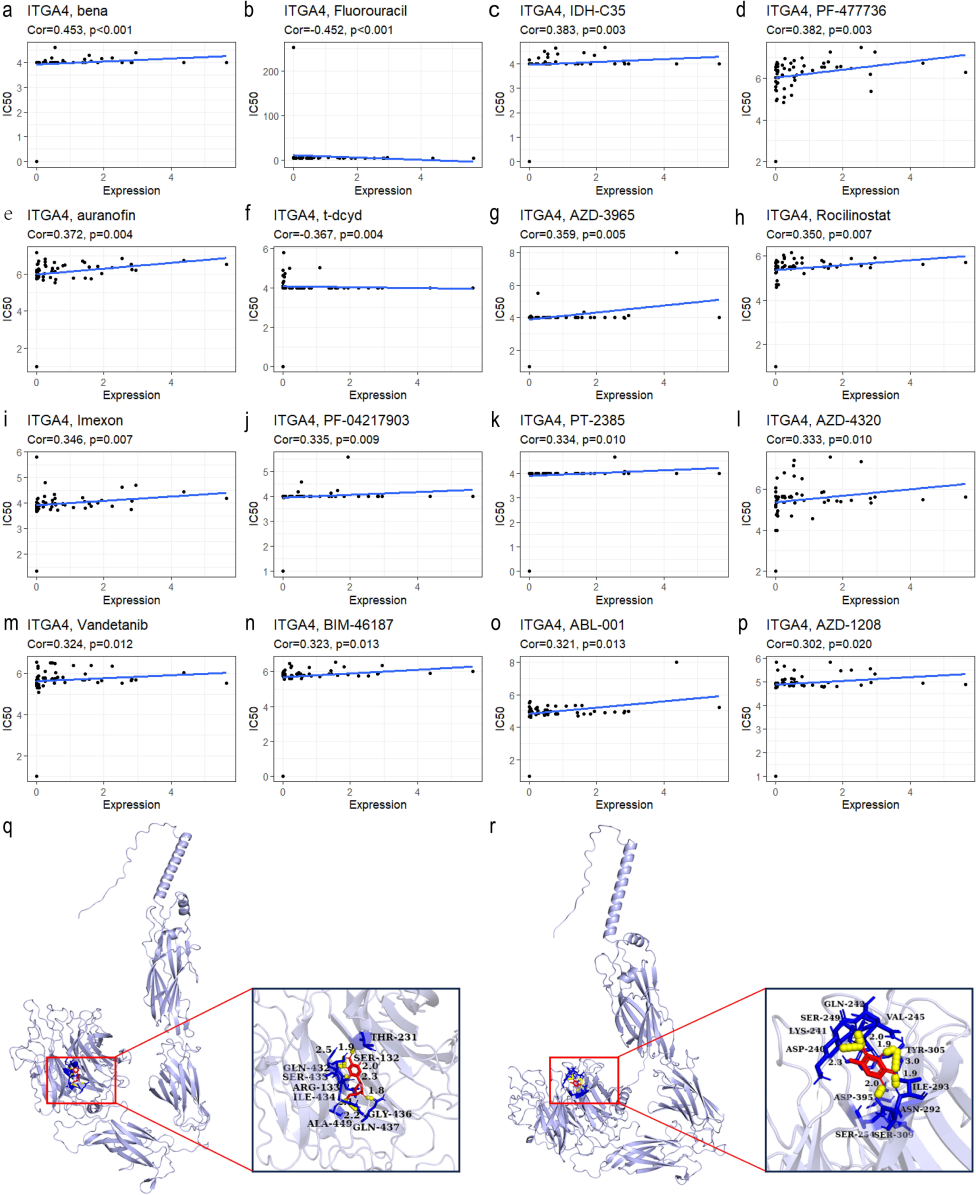
**(A–P)** Top 16 drugs with IC50 values most correlated with ITGA4 expression in the CellMiner database. Molecular docking of t-dcyd **(Q)** and Fluorouracil **(R)** with ITGA4 protein.

**Figures 8A–P** presents the top 16 drugs from the CellMiner database whose IC50 values are most significantly correlated with ITGA4 expression. Specifically, Fluorouracil and t-dcyd exhibit negative correlations with ITGA4 expression levels, with protein docking free energies of -4.53 kcal/mol and -5.47 kcal/mol respectively (**Figures 8Q, R**), indicating that cancers with higher ITGA4 may respond better to these drugs. Conversely, the IC50 values of the other 14 drugs are positively correlated with ITGA4 expression, suggesting potential resistance in tumors with elevated ITGA4 levels. These results underscore ITGA4’s potential impact on drug sensitivity in cancer, proposing it as a viable target for therapeutic intervention.

### 3.8 Validation of pan-cancer insights in GC analysis

**Figure 9:**
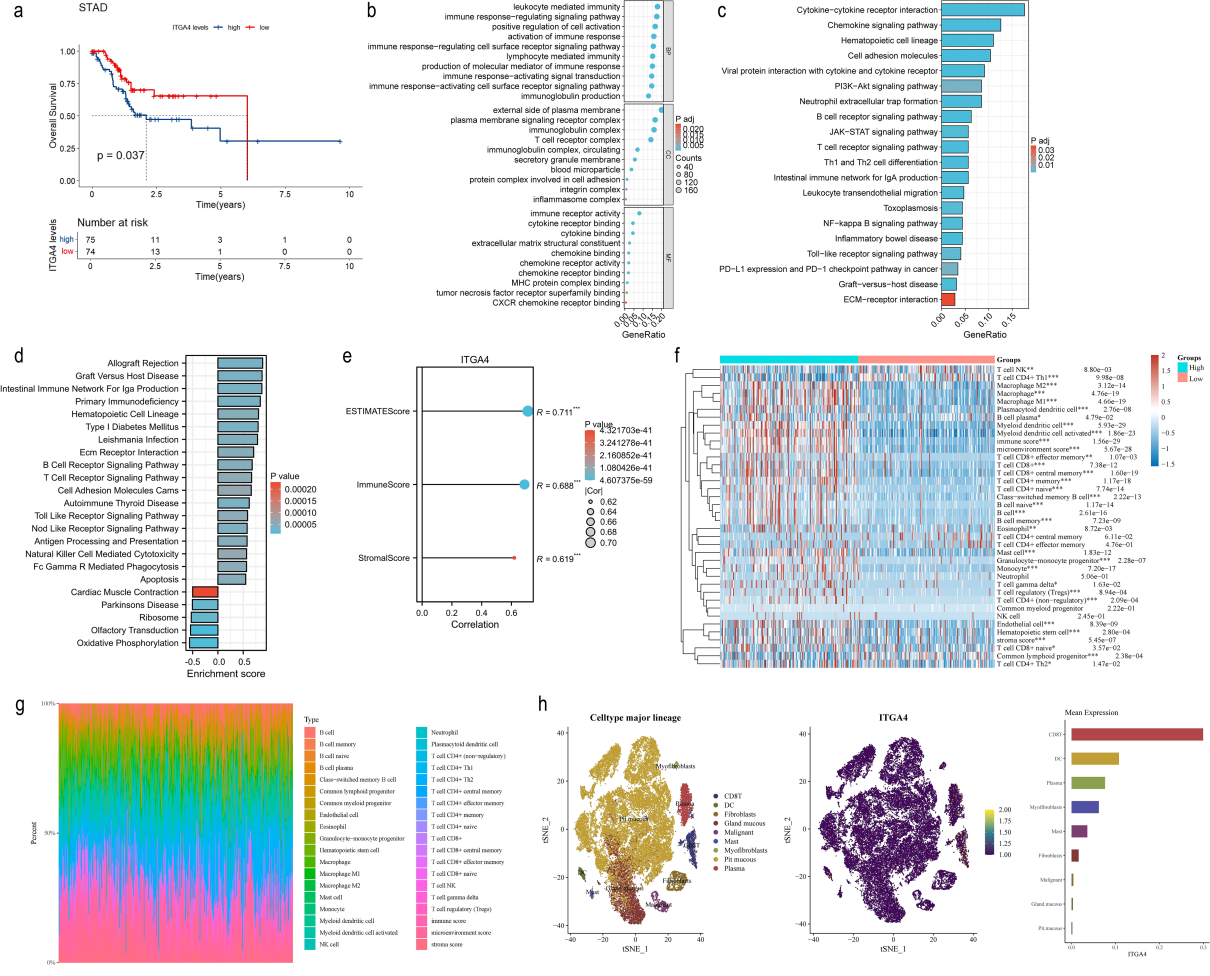
Prognostic value, functional analysis, and immune correlation of ITGA4 in GC. **(A)** K-M survival curves of GC patients. **(B)** GO enrichment analysis of ITGA4 in GC. **(C)** KEGG pathway analysis of ITGA4 in GC. **(D)** GSEA of ITGA4 in GC. **(E)** Correlation analysis of ITGA4 with three TME scores in GC. **(F)** Heatmap of the correlation between ITGA4 and immune cell infiltration based on the xCELL algorithm. **(G)** Immune cell infiltration abundance plot based on the xCELL algorithm. **(H)** Single-cell clustering plot, distribution map of ITGA4 expression in different cells, and abundance plot of ITGA4 expression in different cells, based on the STAD-GSE134520 dataset. (*P < 0.05; **P < 0.01; ***P < 0.001).

To validate the pan-cancer findings of ITGA4, we investigated its role in GC using the TCGA-STAD cohort. We observed a poorer prognosis in patients with top 20% ITGA4 expression compared to those with the bottom 20% (**Figure 9A**), correlating with findings from pan-cancer studies linking higher ITGA4 expression to advanced GC stages and grades. Further, DEGs between high and low ITGA4 expression groups were used for enrichment analysis (**Supplementary Figure S8A**). GO results indicated that ITGA4 primarily contributes to cell adhesion, immune infiltration, and immune regulation in GC, involving processes such as immune response-activating signal transduction, protein complexes involved in cell adhesion, chemokine binding, and MHC protein complex binding (**Figure 9B**). Meanwhile, KEGG and GSEA analyses suggested that ITGA4 might regulate crucial physiological processes beyond the TME of GC, such as ECM receptor interaction, cell adhesion, apoptosis, antigen processing and presentation, and oxidative phosphorylation. Moreover, ITGA4 impacts several classic cancer-related signaling pathways including PI3K-Akt, JAK-STAT, NF-kappa B, and Toll-like receptor pathways (**Figure 9C, D**). Additionally, our investigation into ITGA4’s immunological relevance in GC revealed significant positive correlations between its expression and three TME scores (**Figure 9E**). Also, three different algorithms showed significantly higher immune cell infiltration, such as macrophages, CD4^+^ and CD8^+^ T cells, in the high ITGA4 expression group compared to the low expression group (**Figures 9F, G**; **Supplementary Figures S8B–E**). ITGA4 were also noted primarily expressed in CD8^+^ T cells, DC cells, and plasma cells in two GC single-cell sequencing cohorts (GSE134520 and GSE167297) (**Figure 9H**; **Supplementary Figures S8F, G**), suggesting the expression of ITGA4 in these cells may potentially regulate the TME. These findings are generally consistent with the results from pan-cancer analyses and highlight a unique strong immunological correlation associated with ITGA4 in GC.

### 3.9 Validation of ITGA4 expression, function, and clinical relevance in GC

**Figure 10:**
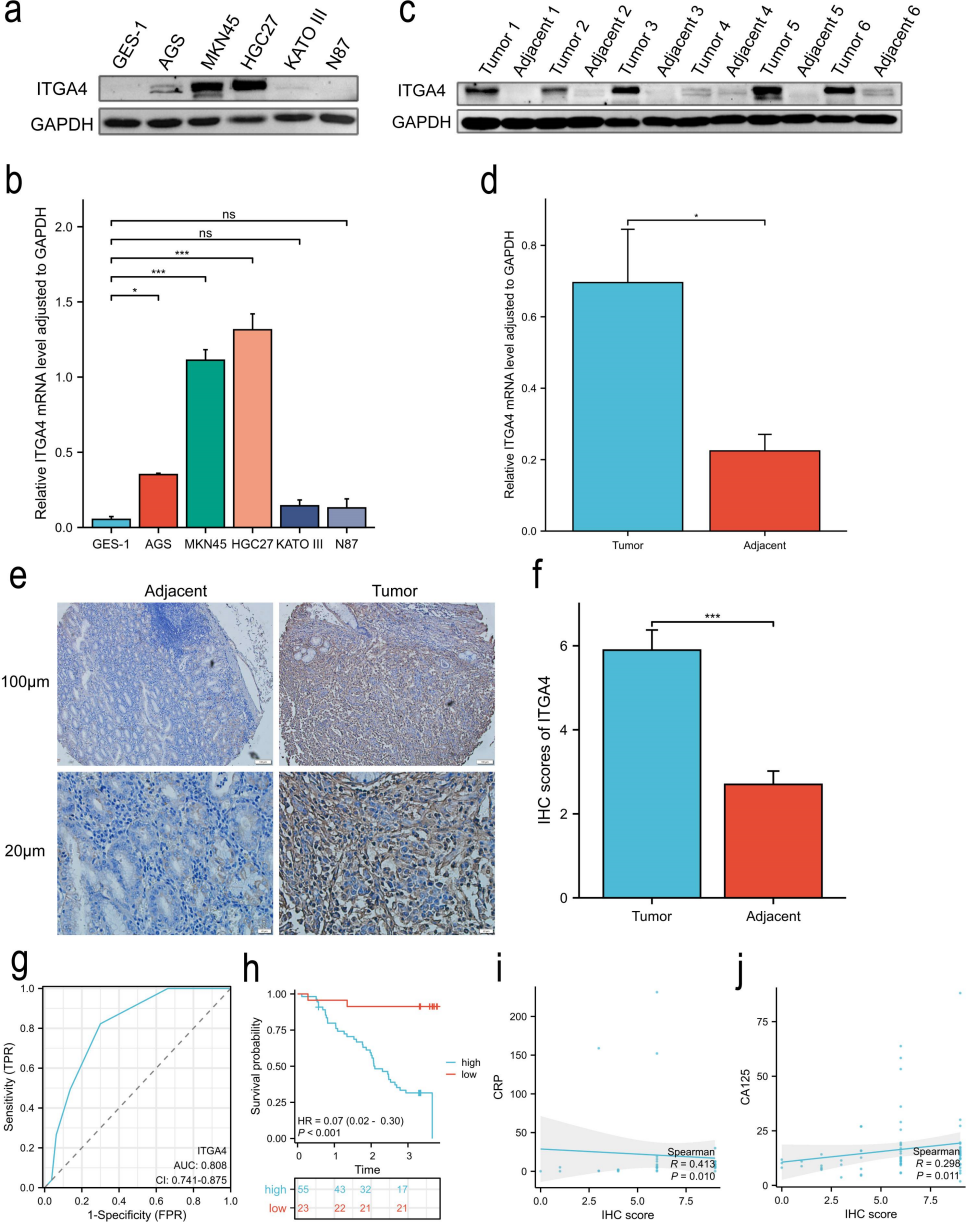
ITGA4 expression, prognostic and diagnostic value, and its correlation with serum biomarkers in GC. **(A, B)** WB analysis of ITGA4 protein expression in a normal gastric epithelial cell line and five GC cell lines. **(C–F)** ITGA4 protein expression in GC tissues and adjacent tissues was analysed through WB (6 pairs) and IHC staining (80 pairs, scale bar, 100 μm and 20 μm). **(G, H)** Diagnostic and prognostic value. **(I, J)** Scatter plots of the correlation between ITGA4 expression and serum CRP and CA125 levels. (*ns*, no significance; *P < 0.05; **P < 0.01; ***P < 0.001). CRP, C-reactive protein; CA125, carbohydrate antigen 125.

WB analysis revealed that ITGA4 expression was significantly higher in GC cell lines AGS, MKN45, and HGC27 compared to the normal GES-1 cell line (**Figures 10A, B**). Similarly, ITGA4 protein levels were elevated in GC tissues relative to adjacent normal tissues (**Figures 10C, D**). IHC analysis of 80 GC cases confirmed these results (**Figures 10E, F**) and showed that ITGA4 protein expression was higher in the low stage GC than in the high stage (**Supplementary Figures S9A, B**). Knockdown of ITGA4 attenuated the GC cell line MKN45 migration and enhanced its apoptosis (**Supplementary Figures S9C–H**). Additionally, ITGA4 expression demonstrated strong diagnostic value for these GC cases (AUC = 0.808), and cases with high ITGA4 expression were associated with poorer OS (**Figures 10G, H**). Furthermore, patients with high ITGA4 expression exhibited more advanced N and pathological stages, increased perineural and vascular invasion (**Table 1**), along with elevated Ki-67 expression (**Supplementary Table S1**). ITGA4 expression was also correlated with blood levels of neutrophils, lymphocytes, and basophils (**Supplementary Table S2**). Moreover, there was a positive correlation between ITGA4 expression and serum levels of C-reactive protein (CRP) and carbohydrate antigen 125 (CA125) (**Figures 10I, J**).

**Table 1:** Association between ITGA4 and the clinicopathological characteristics of GC.

Characteristics	ITGA4		
	High-expression (n=56)	Low-expression (n=24)	*P* value
Sex, n (%)			0.504
Male	39 (50%)	18 (23.1%)	
Female	16 (20.5%)	5 (6.4%)	
Age, mean ± sd	59.091 ± 10.319	61.826 ± 10.765	0.295
T stage, n (%)			0.177
T1+T2	13 (17.6%)	8 (10.8%)	
T3+T4	41 (55.4%)	12 (16.2%)	
N stage, n (%)			**0.012**
N0	11 (14.9%)	10 (13.5%)	
N1+N2	43 (58.1%)	10 (13.5%)	
M stage, n (%)			0.560
M0	51 (68.9%)	20 (27%)	
MX	1 (1.4%)	0 (0%)	
M1	2 (2.7%)	0 (0%)	
Pathological stage, n (%)			**0.043**
I-II	22 (29.7%)	13 (17.6%)	
III-IV	32 (43.2%)	7 (9.5%)	
Perineural invasion, n (%)			**0.006**
+	34 (54%)	7 (11.1%)	
-	11 (17.5%)	11 (17.5%)	
Vascular invasion, n (%)			**0.006**
+	43 (57.3%)	9 (12%)	
-	12 (16%)	11 (14.7%)	
Gastric mucosal hemorrhage/ulcer, n (%)			1.000
+	24 (58.5%)	10 (24.4%)	
-	5 (12.2%)	2 (4.9%)	

The bold text represents values with P < 0.05, indicating that the differences are statistically significant.

## 4 Discussion

Integrins represent attractive targets for the prevention and treatment of malignancies. Given the diverse roles of integrin family members in various cancers (54), developing suitable integrin targets is essential for personalized cancer treatment. Although ITGA4 impacts proliferation, invasion, metastasis, and lymphocyte recruitment of some tumors (16, 17), its specific role in the TME is unclear. Due to the current lack of systematic understanding of ITGA4, we conducted a comprehensive pan-cancer analysis. Our study found that ITGA4 expressed low in most normal tissues, highlighting its potential as a low-toxicity therapeutic target. In contrast to normal tissues, ITGA4 expression significantly differed across various cancer types and different molecular and immune subtypes, suggesting its functional diversity throughout cancers. Besides, higher ITGA4 expression was linked to advanced tumor grades and stages, indicating its role in malignant progression. Additionally, ITGA4 showed good diagnostic potential in 20 cancer types. Prognostic analysis demonstrated that its expression levels correlated with OS, DSS, PFI, and DFI in various cancers, acting as a risk or protective factor, emphasizing the complex role of ITGA4 in cancer prognosis. Predictive nomogram model further validated the clinical decision-making value of ITGA4, showcasing its excellent potential as a tumor biomarker and therapeutic target. As integrin family members play diverse roles in cellular signaling and biological processes, ITGA4 may act as an oncogene in some cancers, promoting tumor cell adhesion, migration, invasion, angiogenesis, and metastasis, or as a tumor suppressor, impacting cell survival, proliferation, and drug response (55). For example, ITGA4 overexpression in chronic lymphocytic leukemia (CLL) correlates with disease progression and high-risk biomarkers (56), while its low expression in colorectal cancer is linked to poor prognosis (57). These expression patterns align with the findings in this study for hematologic malignancies and COADREAD. Based on prior studies, we hypothesize that the differential expression of ITGA4 between may be influenced by: interactions with other molecules or signaling pathways (58); the high heterogeneity of tumors and subtypes (55); mutations in oncogenes and tumor suppressor genes, epigenetic modifications (such as DNA methylation), and transcriptional regulation (59, 60); and changes in the TME (61, 62). To explore the mechanism and clinical significance of ITGA4 in cancer, we conducted a series of subsequent analyses.

Functional enrichment analysis revealed that ITGA4 was involved in cell-substrate adhesion, integrin-mediated cell adhesion, ECM binding, EMT, leukocyte migration, cellular extravasation, cellular quiescence, the cell cycle, DNA damage repair, differentiation, apoptosis, and cancer metastasis, invasion, and angiogenesis. Additionally, ITGA4 regulated multiple canonical cancer signaling pathways, such as PI3K-AKT, RAS-MAPK, RTK, and TSC-mTOR pathways. These results were validated by transcriptome sequencing of GC. Wound-healing assay and flow cytometry also confirmed that ITGA4 downregulation inhibits GC cell migration and promotes apoptosis. Previous study has shown that integrins on the surface of exosomes from tumor cells affect cell adhesion and migration (63). Cell adhesion is linked to proliferation pathways like PI3K/AKT and MEK/ERK pathways, which protect tumor cells from apoptosis (54). Moreover, integrin α4β1 influence the recruitment of inflammatory cells (64). Notably, single-cell analysis of GC in this study revealed that ITGA4 was primarily expressed in CD8^+^ T cells, DC cells, and plasma cells, suggesting its potential regulatory role in TME through these cell types. Therefore, ITGA4 and its mediated signaling pathways play critical roles in the growth, invasion, migration, and immune evasion of malignant tumors. As the role of integrins in the TME is complex and varies according to tumor heterogeneity (65), further research is needed to elucidate the signaling mechanisms related to ITGA4.

Aligning with functional enrichment analysis results, we found that ITGA4 was significantly positively correlated with three TME indicators, suggesting its crucial impact on tumor cells, stromal components, and immune cells. Given the TME’s complex role in tumor malignancy and treatment response (66), targeting ITGA4 to mitigate TME-induced immunosuppression holds promise for improving anti-cancer efficacy. We focused on the immune cell infiltration associated with ITGA4 expression in the overall cancer context. Five algorithms were used to analyze immune cell infiltration based on tumor gene expression profiles and minimize potential biases from individual algorithm. All five algorithms consistently showed that ITGA4 was markedly associated with immune cell infiltration across various tumors, particularly with tumor-associated macrophages (TAMs), M1 and M2 macrophages, and CD4^+^ and CD8^+^ T cells. TAMs can polarize into pro-inflammatory or anti-inflammatory phenotypes, and promote tumor progression and recurrence, leading to poor prognosis and therapeutic resistance (66–69). For instance, M1 macrophages produce pro-inflammatory factors with anti-tumor effects, but under certain conditions, they produce excessive NO, worsening inflammation and contributing to autoimmune diseases like IBD and MS (70, 71). Our findings also showed that ITGA4 was more positively correlated with M2 macrophage abundance than M1 type and negatively correlated with Th1 cells. Since PI3Kγ-integrin α4β1 pathway can reduce IFN-γ levels (72), which is secreted by Th1 cells and enhances M1 macrophage and CD8^+^ T cell activity (73), we hypothesize that ITGA4 could reduce Th1 cells infiltration, leading to the recruitment of less active M1 macrophages and CD8^+^ T cells. Aberrant ITGA4 expression possibly creates an inflammatory environment that polarizes M1 macrophages to M2 macrophages, promoting immunosuppression and recruiting other immune cells to the tumor site (71). Tregs, CAFs, and endothelial cells, which support tumor growth, metastasis, and immunosuppression (74–76), were also significantly positively correlated with ITGA4. These findings were further validated in GC. However, there were some variations in the correlation between ITGA4 expression and immune cell infiltration across different cancer types. For example, all algorithms showed weak or inconsistent correlations in LAML and UCS, contrasting with the positive immune correlations seen in most other tumors. Besides, in the xCELL algorithm, macrophage infiltration in THYM negatively correlated with ITGA4 expression, opposite to the positive correlation in STAD. These differences may stem from tumor microenvironment heterogeneity, immune evasion, immunosuppressive cells, gene regulation, and treatment variations. We further found that ITGA4 was positively correlated with most immune regulatory molecules across cancers, such as CTLA4, PDCD1, LAG3, which inhibit T cell activity. It was also correlated with CSF1R, CCL2, and CCR2, which affect TAM activity (66), indicating ITGA4’s critical role in immune regulation. Therefore, ITGA4 may influence the TME by modulating immune cell infiltration and function in various cancers, where these immune cells have complex roles in promoting or inhibiting tumors, highlighting ITGA4’s potential as a therapeutic target of controlling tumor immune suppression.

The MMR system detects and corrects errors during DNA replication, repair, and recombination (77). Deficiencies in MMR (dMMR) cause high microsatellite instability (MSI-H) and subsequent high tumor mutational burden (TMB-H), resulting in immunogenic neoantigens that improve immune response and prognosis of immunotherapy (78–80). Our study showed a significant positive correlation between ITGA4 and four key MMR components, while ITGA4 expression negatively correlated with MSI and TMB in multiple cancers. Therefore, tumors with low ITGA4 expression may exhibit dMMR, leading to MSI-H and TMB-H phenotypes, which are more responsive to immunotherapy. Meanwhile, ITGA4 showed good predictive value for efficacy in various immunotherapy cohorts. These findings suggest that ITGA4 may be a valuable biomarker for predicting tumor response to immunotherapy, highlighting the potential advantages of screening patients with abnormal ITGA4 expression for personalized immune checkpoint inhibitor (ICI) treatment. Meanwhile, targeting ITGA4 in combination with ICIs may modulate the TME, reduce inflammation and immune evasion, and enhance anti-tumor immune cell infiltration and response. Recent studies suggest that this combination reduces PD-L1 expression in cancer cells and boosts T cell cytotoxicity and infiltration (81). Therefore, ITGA4-targeted drugs combined with immunotherapy may represent an effective cancer treatment strategy.

Genetic alterations of ITGA4, primarily somatic missense mutations, were identified in 22 of 30 cancer types, potentially giving tumors a growth advantage by causing abnormal protein functions (82). Additionally, among the top ten genes with higher mutation frequencies in the ITGA4-altered group, TP53, LRP1B, TTN, MUC16, CSMD3, and SOCS2 have been reported to affect tumor progression, drug resistance, and immunotherapy response (47–50, 52), suggesting that ITGA4 may also be involved in these processes through gene mutation, warranting further research.

The above analysis suggests that ITGA4 may play different roles in different cancers. Methylation analysis showed elevated ITGA4 methylation in several cancers, with a negative correlation between methylation and expression in eight cancer types, and a positive correlation in three. DNA methylation regulates oncogenes and tumor suppressor genes in malignant progression. Tumor heterogeneity leads to varying methylation patterns across cancers, affecting gene expression, classification, treatment response, and prognosis (83–85). Thus, the differential expression of ITGA4 across various cancers may be related to its distinct methylation status in each cancer type. Previous studies support this conclusion. Hypermethylation of CpG sites 1, 2, and 3 in ITGA4 leads to protein dysregulation in CLL and poor prognosis (86). Abnormal methylation of the ITGA4 5′-CpG island can cause expression loss and may predict CHOL metastasis (87). Additionally, methylation of the ITGA4 promoter can suppress its expression in certain tumors (88). However, ITGA4 expression can be enhanced through m6A methylation by METTL3, increasing the homing ability of AML cells, illustrating the complex impact of different methylation states (59). A recent meta-analysis showed that the summary ROC curve’s AUC for ITGA4 methylation across cancers is 0.94 (89), highlighting its strong potential as a diagnostic marker. So, ITGA4 methylation is associated with cancer development and progression and has potential as a biomarker for early cancer screening.

ECM can initiate or promote EMT, enhancing the invasion, metastasis, stemness, and chemoresistance of malignant tumor cells (90–94). Our study implies that ITGA4 may be involved in ECM binding and EMT, and is associated with stemness scores and resistance to various chemotherapeutic agents. Thus, combining ITGA4 inhibitors with chemotherapy may reduce resistance, enhance drug penetration, and improve efficacy while reducing side effects by modulating TME and stemness (81).

We also analyzed the clinical relevance of ITGA4 in 80 GC patients. Ki-67 is a key marker of tumor cell proliferation and has been shown to be associated with pathological staging, infiltration, metastasis, chemotherapy resistance, and prognosis in GC (95, 96). CA125 is a recognized serum tumor marker and has been reported to affect the prognosis of GC patients (97). CRP, an acute-phase protein, reflects the inflammatory response (98). This study revealed that GC patients with high ITGA4 expression exhibited advanced N and pathological stages, stronger perineural and vascular invasion, along with higher Ki-67 expression and poorer prognosis. Additionally, ITGA4 expression was positively correlated with serum levels of CA125 and CRP, and related to blood immune cell abundance. These findings suggest that ITGA4 may promote GC cell proliferation and disease progression, while also participating in inflammatory responses, ultimately leading to unfavorable outcomes. Monitoring serum levels of CA125 and CRP to assess the potential role of ITGA4 in GC may provide new insights for personalized precision therapies, warranting further investigation in future studies.

This study systematically analyzed the role of ITGA4 in pan-cancer and validated some of its functions through experiments. Some issues should be pointed. In WB experiment, the appearance of double bands in some samples could be due to several factors: ITGA4 protein may exist in isoforms or undergo post-translational modifications, the antibody may cross-react with similar proteins, alternative splicing may produce different isoforms, protein degradation may lead to smaller products, or antibody specificity issues may cause non-specific binding. Additionally, future research should include deeper cell and animal studies to clarify ITGA4 mechanisms in cancer, along with larger-scale data analyses for cancer types with small sample sizes. Furthermore, we validated ITGA4 in GC only, emphasizing the need for further research across other cancer types. Notably, our study identified ITGA4 as a potential prognostic and immunotherapeutic biomarker, which holds bidirectional implications, highlighting the importance to consider its diverse expression and functions across tumor types. Besides, translating current research into clinical applications faces challenges, including the complex differences between cell and animal models, insufficient pharmacokinetic and pharmacodynamic studies, variability in target expression and function due to tumor heterogeneity, difficulties in customizing personalized treatment strategies, challenges in patient recruitment and stratification for clinical trials, the complexity of selecting clinical endpoints, potential risks of long-term drug use, and adverse reactions and management (55). These challenges require comprehensive and long-term efforts to address.

## 5 Conclusion

To sum up, ITGA4 and its associated signaling pathways are critical in tumor growth, invasion, metastasis, immune regulation, heterogeneity, stemness, chemoresistance, and immunotherapy response, making it a promising biomarker and therapeutic target. Combining ITGA4-targeted therapies with traditional or immunotherapies may enhance clinical outcomes. Besides, in GC cases, ITGA4 promotes tumor cell proliferation, invasion and metastasis, while also contributing to inflammatory responses and leading to adverse outcomes. Further studies are required to elucidate more molecular mechanisms of ITGA4 to advance cancer diagnosis and treatment.

## Data Availability

The original contributions presented in the study are included in the article/**Supplementary Material**. Further inquiries can be directed to the corresponding authors.
