# Supplementary material for: ITGA4 as a potential prognostic and immunotherapeutic biomarker in human cancer and its clinical significance in gastric cancer: an integrated analysis and validation
Source: Front Oncol. 2025 Feb 12;15:1513622. doi: 10.3389/fonc.2025.1513622 (PMC11860100; doi:10.3389/fonc.2025.1513622)
Supplement: Supplementary file 1 [file DataSheet1.docx]

Supplementary Material

# Supplementary Figures and Tables

## Supplementary Figures

**
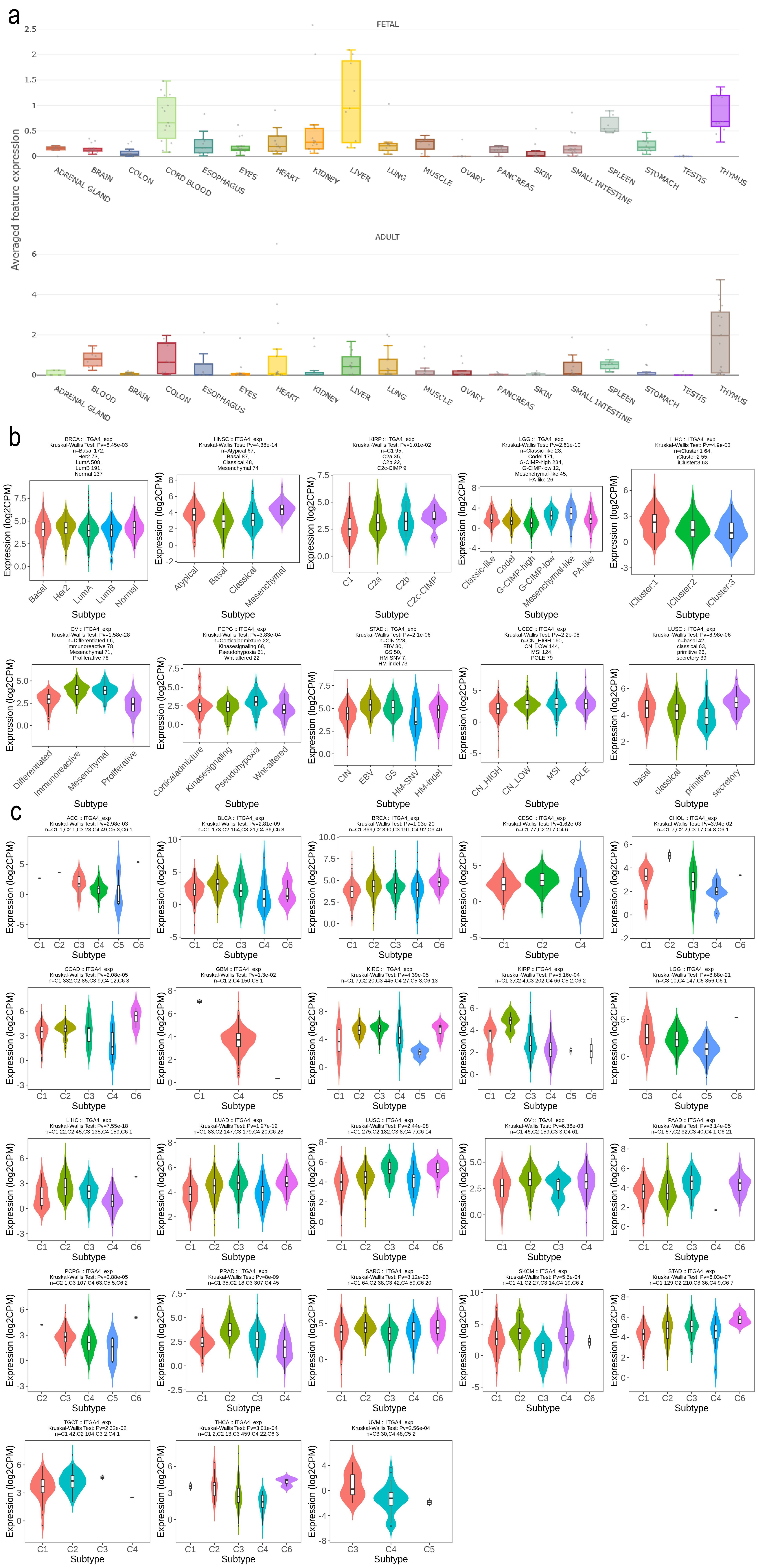
**

**Figure S1.** Expression of ITGA4 in normal tissues and tumor Subtypes. (a) Expression of ITGA4 in normal tissues of infants and adults from the HTCA database. (b) Expression of ITGA4 among various molecular subtypes. (c) Expression of ITGA4 among different immune subtypes.


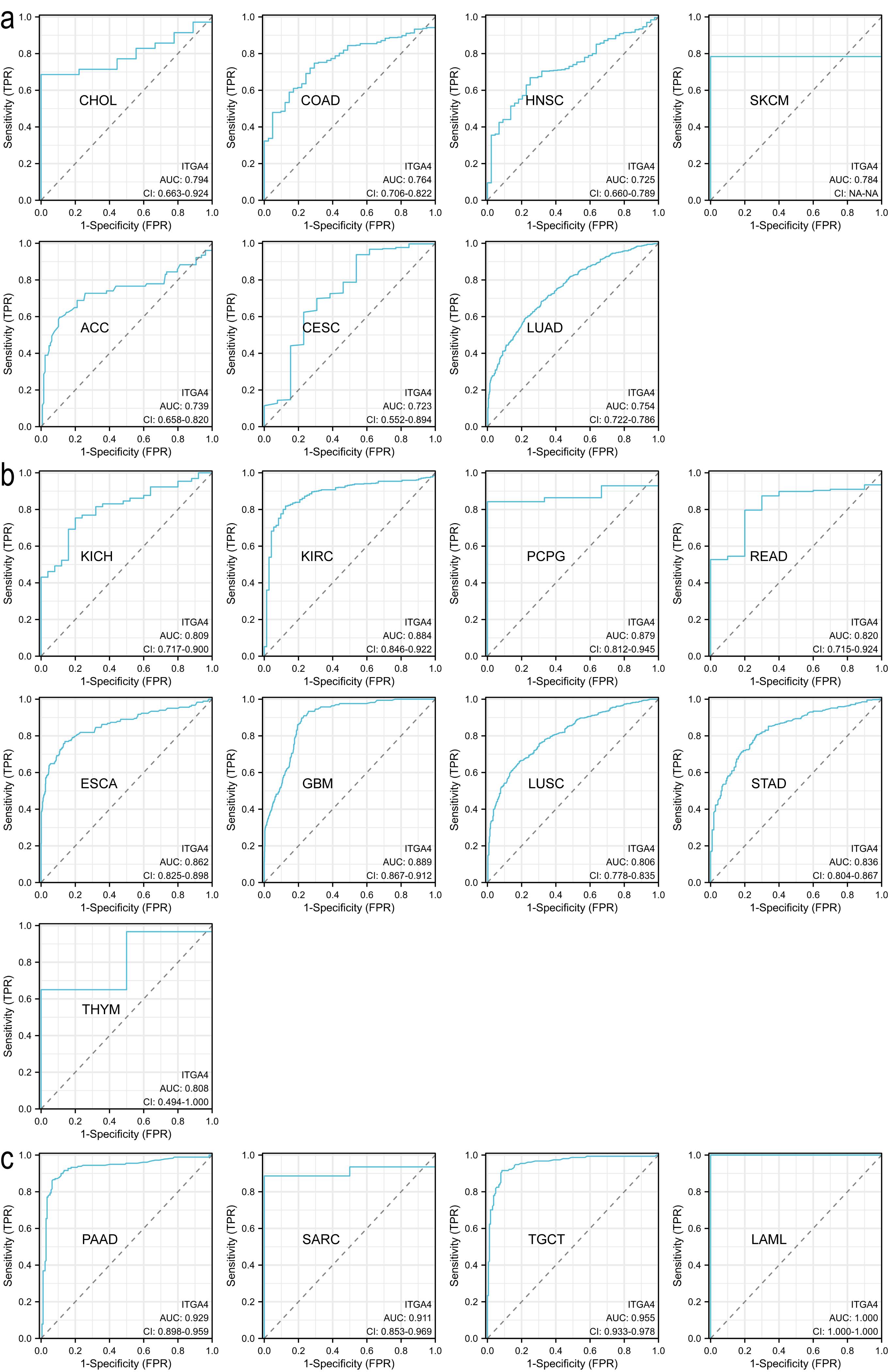


**Figure S2.** Diagnostic value of ITGA4. (a) Cancer types with the AUC levels between 0.7 and 0.8. (b) Cancer types with an AUC levels between 0.8 and 0.9. (c) Cancer types with an AUC levels greater than 0.9.





**Figure S3.** K-M survival curves for high and low ITGA4 Expression Groups Across Different Cancer Types. (a) OS in six cancer types. (b) DSS in seven cancer types. (c) DFI in three cancer types. (d) PFI in four cancer types.


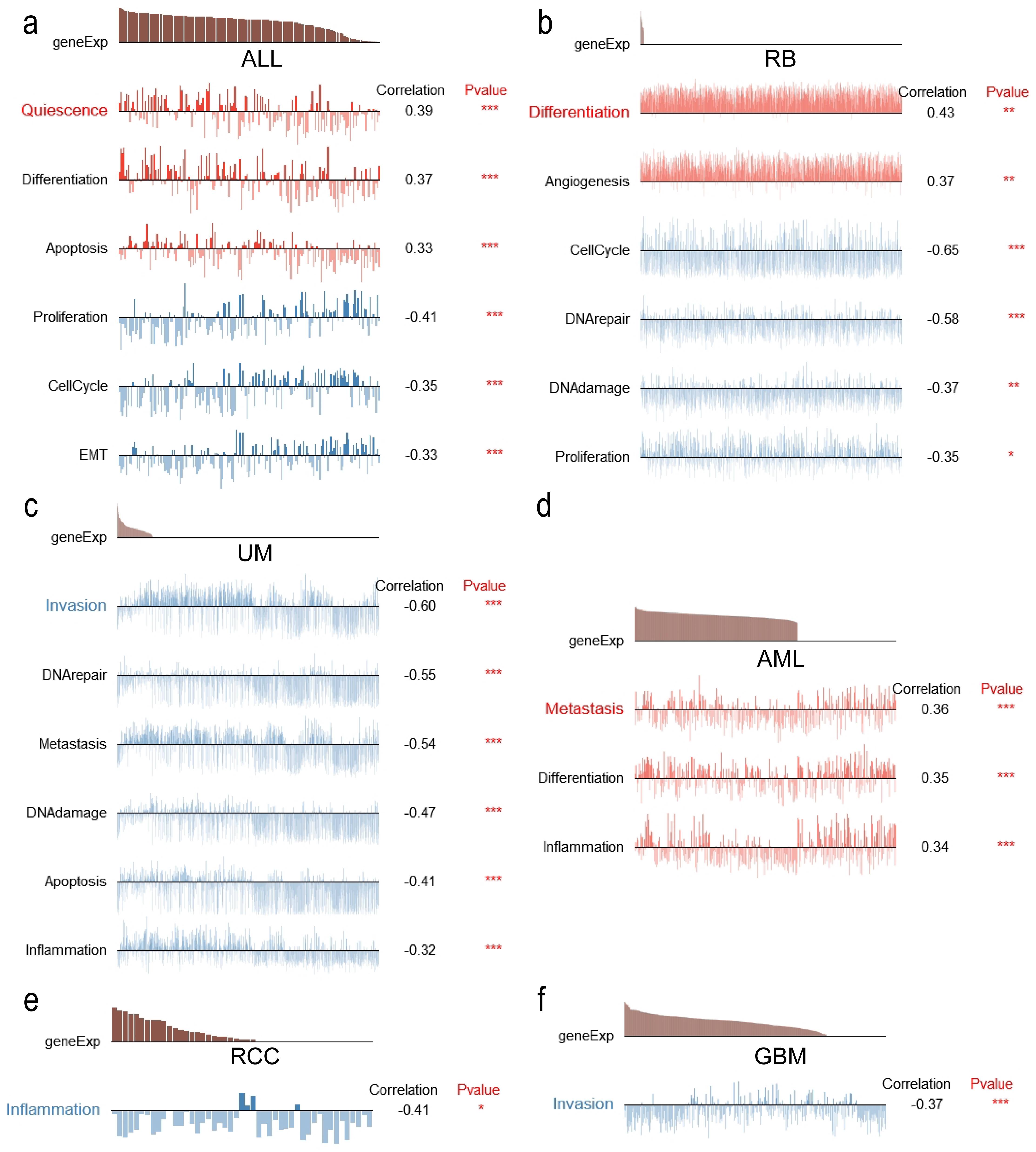


**Figure S4.** Single-cell functional analysis of ITGA4 in ALL (a), RB (b), UM (c), AML (d), RCC (e), and GBM (f) using the CancerSEA database. **P* < 0.05; ***P* < 0.01; ****P* < 0.001.


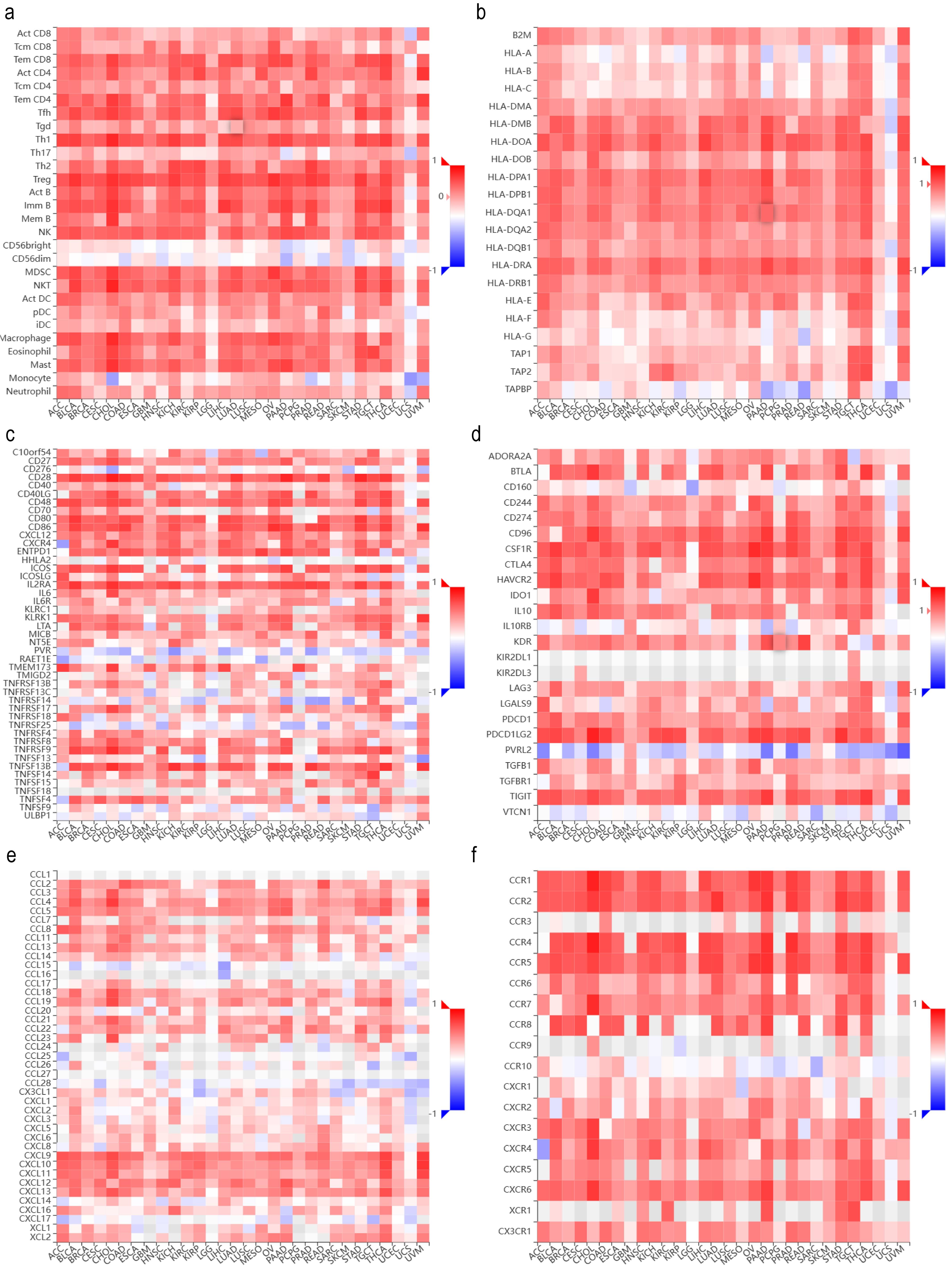


**Figure S5.** Relationship between ITGA4 and tumor Immune regulation. Heatmaps from the TISIDB database showing the correlation of ITGA4 with tumor-infiltrating lymphocytes (a), MHC molecules (b), immunostimulators (c), immunoinhibitors (d), chemokines (e), and chemokine receptors (f). **P* < 0.05; ***P* < 0.01; ****P* < 0.001.


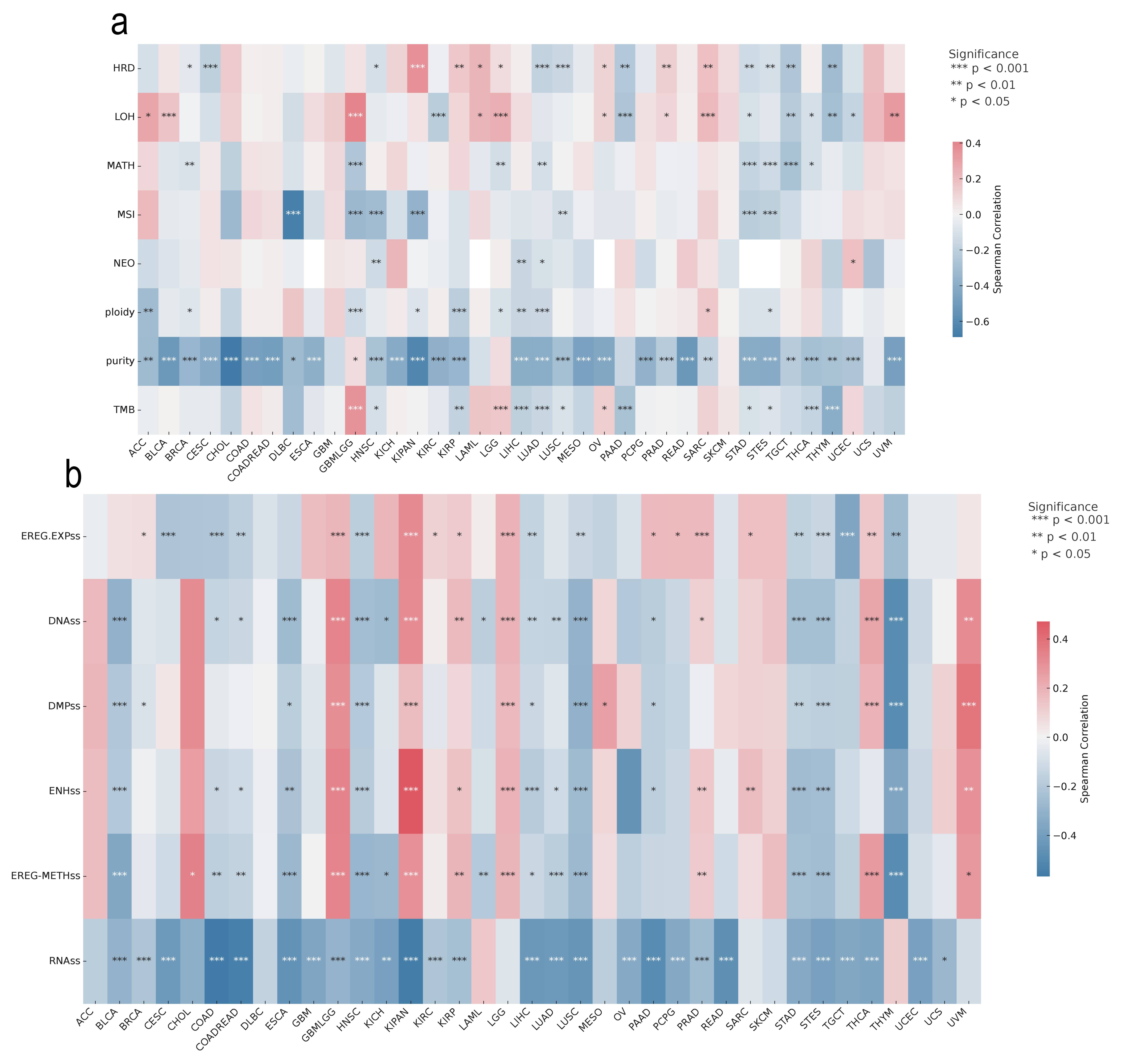


**Figure S6.** ITGA4’s relationship with tumor heterogeneity and stemness. (a) Heatmap of the correlation between ITGA4 and eight tumor heterogeneity parameters. (b) Heatmap of the correlation between ITGA4 and six tumor stemness parameters. **P* < 0.05; ***P* < 0.01; ****P* < 0.001.





**Figure S7.** ITGA4 alteration distribution and specific site methylation levels in pan-cancer. (a)The distribution of ITGA4 alterations across various cancer types. (b) Methylation levels of 18 specific ITGA4 sites in pan-cancer and normal tissues. *ns*, no significance; **P* < 0.05; ***P* < 0.01; ****P* < 0.001.


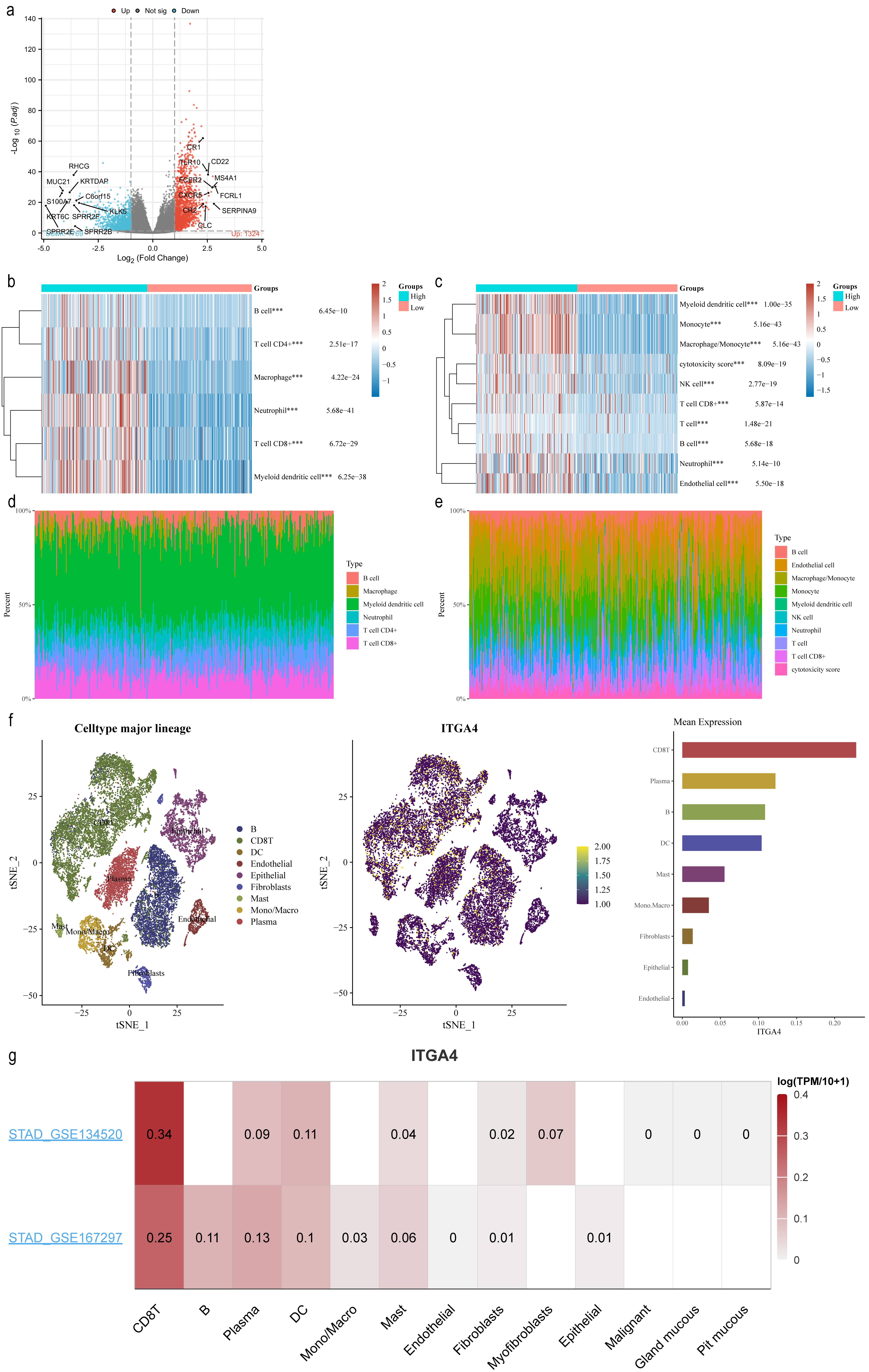


**Figure S8.** Immune correlation analysis of ITGA4 in GC. (a) Volcano plot of DEGs between high and low ITGA4 expression groups. (b, d) Heatmap of the correlation between ITGA4 and immune cell infiltration and the immune cell infiltration abundance plot based on the TIMER algorithm. (c, e) Heatmap of the correlation between ITGA4 and immune cell infiltration and the immune cell infiltration abundance plot based on the MCPCounter algorithm. (f) Single-cell clustering plot, distribution map of ITGA4 expression in different cells, and abundance plot of ITGA4 expression in different cells based on the STAD-GSE167297 dataset. (g) Heatmap of ITGA4 expression in different cells from the GSE134520 and GSE167297 datasets. **P* < 0.05; ***P* < 0.01; ****P* < 0.001.





**Figure S9.** Functional validation of ITGA4 and its expression differences in high- and low-stage GC. (a, b) IHC analysis of ITGA4 expression in low- and high-stage GC and statistical analysis. (c, d) WB validation of ITGA4 knockdown efficiency in MKN45 cell line. (e, f) Wound-healing assay results and corresponding statistical analysis of MKN45 cells. (g, h) Flow cytometry analysis and statistical analysis of MKN45 cells. **P* < 0.05; ***P* < 0.01; ****P* < 0.001.

- 1. **Supplementary Tables**

**Table S1** Association between ITGA4 and the tumor biomarkers of GC.

| **Tumor biomarkers** | **ITGA4** | | |
| --- | --- | --- | --- |
|  | **High-expression (n=56)** | **Low-expression (n=24)** | ***P* value** |
| CKp, n (%) |  |  | 0.162 |
| + | 46 (69.7%) | 18 (27.3%) |  |
| - | 0 (0%) | 2 (3%) |  |
| CK8/18, n (%) |  |  | 1.000 |
| + | 53 (70.7%) | 21 (28%) |  |
| - | 1 (1.3%) | 0 (0%) |  |
| P53, n (%) |  |  | 0.580 |
| mutant-type | 7 (50%) | 2 (14.3%) |  |
| wild-type | 3 (21.4%) | 2 (14.3%) |  |
| C-erbB-2, n (%) |  |  | 0.792 |
| - | 42 (57.5%) | 15 (20.5%) |  |
| + | 6 (8.2%) | 2 (2.7%) |  |
| 2+ | 5 (6.8%) | 3 (4.1%) |  |
| LMP-1, n (%) |  |  | 0.950 |
| - | 36 (69.2%) | 14 (26.9%) |  |
| + | 2 (3.8%) | 0 (0%) |  |
| MLH-1, n (%) |  |  | 1.000 |
| + | 48 (67.6%) | 18 (25.4%) |  |
| - | 4 (5.6%) | 1 (1.4%) |  |
| PMS-2, n (%) |  |  | 0.694 |
| + | 47 (69.1%) | 18 (26.5%) |  |
| - | 3 (4.4%) | 0 (0%) |  |
| Ki-67, median (IQR) | 70 (60, 70) | 60 (40, 70) | **0.040** |
| Syn, n (%) |  |  | 0.658 |
| - | 44 (60.3%) | 15 (20.5%) |  |
| + | 9 (12.3%) | 5 (6.8%) |  |

**Table S2** Association between ITGA4 and immune cell blood levels of GC.

| **Immune cells** | **ITGA4** | | |
| --- | --- | --- | --- |
|  | **High-expression （n=56）** | **Low-expression （n=24）** | ***P* value** |
| Leukocyte, median (IQR) | 5.64 (4.88, 7.19) | 7 (5.25, 9.545) | 0.136 |
| Neutrophil count, median (IQR) | 3.82 (2.545, 4.98) | 4.69 (3.18, 8.42) | **0.075** |
| Neutrophil percentage, mean ± sd | 0.64945 ± 0.13962 | 0.73565 ± 0.14532 | **0.016** |
| Lymphocyte count, median (IQR) | 1.32 (1.06, 1.76) | 0.91 (0.68, 1.425) | **0.029** |
| Lymphocyte percentage, median (IQR) | 0.23 (0.19, 0.32) | 0.17 (0.065, 0.285) | **0.019** |
| Eosinophil count, median (IQR) | 0.07 (0.03, 0.165) | 0.07 (0.01, 0.13) | 0.432 |
| Eosinophil percentage, median (IQR) | 0.01 (0.01, 0.03) | 0.01 (0, 0.025) | 0.264 |
| Basophil count, median (IQR) | 0.03 (0.02, 0.035) | 0.02 (0.01, 0.03) | **0.048** |
| Basophil percentage, median (IQR) | 0.01 (0, 0.01) | 0 (0, 0.01) | **0.019** |
| Monocyte count, median (IQR) | 0.38 (0.295, 0.48) | 0.44 (0.335, 0.515) | 0.224 |
| Monocyte percentage, median (IQR) | 0.07 (0.05, 0.09) | 0.07 (0.045, 0.08) | 0.855 |

- 1. **Abbreviation**

| ACC | Adrenocortical Carcinoma |
| --- | --- |
| ALL | Acute Lymphoblastic Leukemia |
| BLCA | Bladder Urothelial Carcinoma |
| BRCA | Breast Invasive Carcinoma |
| CESC | Cervical Squamous Cell Carcinoma and Endocervical Adenocarcinoma |
| CHOL | Cholangiocarcinoma |
| COAD | Colon Adenocarcinoma |
| DLBC | Diffuse Large B-Cell Lymphoma |
| ESCA | Esophageal Carcinoma |
| GBM | Glioblastoma Multiforme |
| HNSC | Head and Neck Squamous Cell Carcinoma |
| KICH | Kidney Chromophobe |
| KIRC | Kidney Renal Clear Cell Carcinoma |
| KIRP | Kidney Renal Papillary Cell Carcinoma |
| KIPAN | Pan-kidney Cohort |
| LAML | Acute Myeloid Leukemia |
| LGG | Lower Grade Glioma |
| LIHC | Liver Hepatocellular Carcinoma |
| LUAD | Lung Adenocarcinoma |
| LUSC | Lung Squamous Cell Carcinoma |
| MESO | Mesothelioma |
| OV | Ovarian Serous Cystadenocarcinoma |
| PAAD | Pancreatic Adenocarcinoma |
| PCPG | Pheochromocytoma and Paraganglioma |
| PRAD | Prostate Adenocarcinoma |
| READ | Rectum Adenocarcinoma |
| SARC | Sarcoma |
| SKCM | Skin Cutaneous Melanoma |
| STAD | Stomach Adenocarcinoma |
| STES | Stomach and Esophageal Carcinoma |
| TGCT | Testicular Germ Cell Tumors |
| THCA | Thyroid Carcinoma |
| THYM | Thymoma |
| UCEC | Uterine Corpus Endometrial Carcinoma |
| UCS | Uterine Carcinosarcoma |
| UVM | Uveal Melanoma |
| WT | Wilms Tumor |
